# Distinct Microbial Signatures Associated With Different Breast Cancer Types

**DOI:** 10.3389/fmicb.2018.00951

**Published:** 2018-05-15

**Authors:** Sagarika Banerjee, Tian Tian, Zhi Wei, Natalie Shih, Michael D. Feldman, Kristen N. Peck, Angela M. DeMichele, James C. Alwine, Erle S. Robertson

**Affiliations:** ^1^Tumor Virology Program, Department of Otorhinolaryngology-Head and Neck Surgery and Microbiology, Abramson Cancer Center, Perelman School of Medicine, University of Pennsylvania, Philadelphia, PA, United States; ^2^Department of Computer Science, New Jersey Institute of Technology, Newark, NJ, United States; ^3^Department of Pathology and Laboratory Medicine, Perelman School of Medicine, University of Pennsylvania, Philadelphia, PA, United States; ^4^Division of Hematology Oncology, Department of Medicine, Perelman School of Medicine, University of Pennsylvania, Philadelphia, PA, United States; ^5^Department of Cancer Biology, Perelman School of Medicine, University of Pennsylvania, Philadelphia, PA, United States

**Keywords:** microbiome, endocrine receptor positive breast cancer, triple negative breast cancer, triple positive breast cancer, HER2 positive breast cancer

## Abstract

A dysbiotic microbiome can potentially contribute to the pathogenesis of many different diseases including cancer. Breast cancer is the second leading cause of cancer death in women. Thus, we investigated the diversity of the microbiome in the four major types of breast cancer: endocrine receptor (ER) positive, triple positive, Her2 positive and triple negative breast cancers. Using a whole genome and transcriptome amplification and a pan-pathogen microarray (PathoChip) strategy, we detected unique and common viral, bacterial, fungal and parasitic signatures for each of the breast cancer types. These were validated by PCR and Sanger sequencing. Hierarchical cluster analysis of the breast cancer samples, based on their detected microbial signatures, showed distinct patterns for the triple negative and triple positive samples, while the ER positive and Her2 positive samples shared similar microbial signatures. These signatures, unique or common to the different breast cancer types, provide a new line of investigation to gain further insights into prognosis, treatment strategies and clinical outcome, as well as better understanding of the role of the micro-organisms in the development and progression of breast cancer.

## Introduction

Breast cancer, the second leading cause of cancer death in women, is responsible for the death of 1 in 52 women below 50 years of age (American Cancer Society, 2017). The American Cancer Society estimated that there will be 255,180 new breast cancer cases (2,470 men and 252,710 women) in the US by 2017 (American Cancer Society, 2017). Based on the immuno histochemical classification of hormone receptor status in the cancerous breast cells, there are 4 major groups of breast cancers: endocrine receptor (estrogen or progesterone receptor) positive (abbreviated in the study as BRER), human epidermal growth factor receptor 2 (HER2) positive (abbreviated in the study as BRHR), triple positive (estrogen, progesterone and HER2 receptor positive) (abbreviated in the study as BRTP) and triple negative (absence of estrogen, progesterone and HER2 receptors) (abbreviated in the study as BRTN) (Schnitt, 2010; American Cancer Society, 2017). These four types have specific prognoses and responses to therapy. Specifically, the hormone receptor positive breast cancers (BRER, BRTP) respond to endocrine therapy and show better prognosis, while the hormone receptor negative types (BRHR, BRTN) are more aggressive, non-responsive to endocrine therapy and have poor prognosis (Schnitt, 2010). BRTN cancer is seen in 15–20% of breast cancer patients, is the most aggressive of all the breast cancers, is unresponsive to treatment, highly angiogenic, proliferative and has the lowest survival rate (Siegel et al., 2016).

However in the recent times the global gene expression studies have identified breast tumors further into distinct molecular classes based on the expression level of endocrine receptors, proliferative genes, oncogenes and other genes; luminal A (ER+/PR+ and Ki67 high), luminal B (ER+/PR+, Ki67 low or, ER+/PR+/HE R2+), HER2+, basal (ER-/PR-/basal myoepithelial markers high/ EGFR+), and normal breast-like (ER-/PR-/basal myoepithelial markers-/EGFR-) (Yersal and Barutca, 2014).

Among the risk factors to develop cancer in general, infectious agents are known to be the third highest after tobacco usage and obesity, contributing 15–20% of cancer incidence (Morales-Sanchez and Fuentes-Panana, 2014). Age and genetic pre-disposition are also known cancer risk factors; however, the majority of cancers have unknown etiology (Madigan et al., 1995). Recent studies of microbiome dysbiosis in human health suggest specific changes in the microbiome in a number of disease states (Turnbaugh et al., 2006; Xuan et al., 2014; Chen and Wei, 2015), including cancer (Sheflin et al., 2014; Xuan et al., 2014). Further, studies have suggested the association of particular micro-organisms with specific cancers (Banerjee et al., 2015, 2017a,b; Chen and Wei, 2015). Thus, a distinct microbiome may contribute to the cause or development of cancer. Conversely, the tumor micro-environment may provide a specialized niche in which these viruses and microorganisms may persist. In either case, cancer-type specific microbial signatures may provide clues for early diagnosis, prognosis and the design of treatment strategies.

We have recently identified a distinct microbial signature associated with triple negative breast cancer (Banerjee et al., 2015). In the present study we asked whether the microbial signatures associated with BRTN are shared by other breast cancer types, or do different breast cancer types have unique signatures. To study this we screened BRTN, BRTP, BRER, and BRHR samples using PathoChip, a pan-pathogen array containing oligonucleotide probes for the detection of all known, sequenced viruses, as well as known human bacterial, parasitic, and fungal pathogens. Additionally, PathoChip contains viral family specific conserved probes that allow for detection of uncharacterized members of the viral families (Baldwin et al., 2014). The PathoChip screen includes a whole genome and transcriptome amplification step that allowed detection of very low copy number of both DNA and RNA viruses and micro-organisms from the cancer tissues (Baldwin et al., 2014; Banerjee et al., 2015). Our analyses now show distinct microbial signatures for BRTN and BRTP samples, while the BRER and BRHR samples had similar microbial signatures.

## Materials and methods

### Study samples

The study was approved by the institutional review board at the University of Pennsylvania (Protocol number 819358). All methods were performed in accordance with the relevant guidelines and regulations and reviewed by resident pathologists at the UPENN hospital. In the present study, 50 endocrine receptor (estrogen or progesterone receptor) positive (abbreviated as BRER in the study), 34 human epidermal growth factor receptor 2 (HER2) positive (abbreviated as BRHR in the study), 24 triple positive (estrogen, progesterone and HER2 receptor positive) (abbreviated as BRTP in the study) and 40 triple negative (absence of estrogen, progesterone and HER2 receptors, abbreviated as BRTN in the study) breast cancer tissues were included along with 20 breast control samples from healthy individuals. Due to HIPAA regulations, we could not obtain any information regarding the type of treatment these breast cancer patients received, or, if they were new patients. These tissues were obtained as de-identified archived samples. Tumors needing macro-dissection were received in the form of 10 μm sections on glass slides with marked guiding H&E slides, while tumors that did not require macro-dissection were received as 10 μm paraffin rolls. The 20 non-matched control tissues were derived from breast reduction surgeries and obtained as 10 μm paraffin rolls. Our resident pathologist reviewed case history, confirmed the tumor types and demarcated the cancer cells. All the samples were de-identified FFPE (formalin fixed paraffin embedded) samples of breast tumors or controls, and were received from the Abramson Cancer Center Tumor Tissue and Biosample Core. Extreme care was taken to avoid contamination during cutting of the FFPE sections (Banerjee et al., 2015). For each samples, microtome and other equipments were cleaned with 70% ethanol. Further, a new blade was used to prepare and cut each sample, and the area was also de-contaminated before cutting each sample (Banerjee et al., 2015).

### Pathochip design, sample preparation, and microarray processing

The PathoChip Array design has been previously described in detail (Baldwin et al., 2014). The PathoChip probes were generated *in silico* using the genome sequences of all known viruses, as well as known human bacterial, parasitic and fungal pathogens. The PathoChip comprises 60,000 probe sets manufactured as SurePrint glass slide microarrays (Agilent Technologies Inc.), containing 8 replicate arrays per slide. Each probe is a 60-nt DNA oligomer that targets multiple genomic regions of the micro-organisms, for example, 18S rRNA gene, 5.8S rRNA gene, 28S rRNA gene, ITS1 and ITS2 for parasite detections, 16S rRNA gene for bacteria detections, 18S rRNA gene, ITS1, 5.8S rRNA gene, ITS2 and 26S rRNA gene to detect fungi, and conserved and specific viral genes to detect viral families and specific viruses. PathoChip screening was done using both DNA and RNA extracted from formalin-fixed paraffin-embedded (FFPE) tumor tissues as described previously (Baldwin et al., 2014; Banerjee et al., 2015). The quality of the extracted nucleic acids was determined by agarose gel electrophoresis and the A260/280 ratio. The extracted RNA and DNA were subjected to whole genome and transcriptome co-amplification (WTA) as previously described (Banerjee et al., 2015). A non-template control (RNAase/DNAase free water) was used during the WTA step, to determine if any contamination was present during the amplification step. The quality of the WTA products was determined by agarose gel electrophoresis. Human reference RNA and DNA were also extracted from the human B cell line, BJAB and were used for WTA as previously described (Banerjee et al., 2015). The WTA products were purified, (PCR purification kit, Qiagen, Germantown, MD, USA); the WTA products from the cancers were labeled with Cy3 and those from the human reference DNA were labeled with Cy5 (SureTag labeling kit, Agilent Technologies, Santa Clara, CA). The labeled DNAs were purified and hybridized to the PathoChip as described previously (Banerjee et al., 2015). Post-hybridization, the slides were washed, scanned and visualized using an Agilent SureScan G4900DA array scanner (Banerjee et al., 2015).

The question of potential contamination of FFPE blocks or during processing is always a concern. In these experiments all samples were handled and processed in the pathology laboratory using standard aseptic conditions. Likewise the preparation of the DNA and RNA from the samples was done in a dedicated laboratory under established condition designed to minimize laboratory contamination.

### Microarray data extraction and statistical analysis

Agilent Feature Extraction software (Baldwin et al., 2014; Banerjee et al., 2015) was used to extract the raw data from the microarray images. We used the R program for normalization and data analyses (R Core Team, 2015). We calculated scale factor using the signals of green (Cy3) and red (Cy5) channels for human probes. Scale factors are the sum of green/sum of red signal ratios of human probes. Then we used scale factors to obtain normalized signals for all other probes. For all probes except human probes, normalized signal is log2 transformed of green signals/scale factors modified red signals (log2 g – scale factor ^*^ log2 r). On the normalized signals, one-sided *t*-test is applied to select probes significantly present in cancer samples by comparing cancer samples vs. controls. The significance cut-off was log2 fold change of signal ≥1 and adjusted *p*-value (all *p*-value were adjusted via Benjamini–Hochberg procedure for controlling FDR) ≤0.01, control prevalence ≤25%, case prevalence ≥40%. Prevalence is calculated as the detection of the microbial signatures in the cancer and in the control samples as percentages. For a particular microbial signature with multiple probes, we calculated the prevalence of that signature by calculating the maximum number of samples that contained even one of the probes of that signature.

The cancer samples were also subjected to hierarchical clustering, based on the detection of microbial signatures in the samples. We used hierarchical clustering technic (Euclidean distance, complete linkage, normalized hybridization signals not scaled) to cluster samples which were represented as heatmaps (Kolde, 2015). Then clusters were further validated by CH- index (Calinski and Harabasz index) which is implemented in the R package as NbClust (Charrad et al., 2014). CH- index is a cluster index that maximizes inter-cluster distances and minimizes intra-cluster distances. We calculated the possible cluster solution that would maximize the index values to achieve the best clustering of the data. Statistical significance between different groups was determined using the two-sided *t*-test.

Based on the clinical outcomes of the different breast cancer patients, the cases for each breast cancer types were divided into two groups: alive and deceased (with severe outcomes) (Supplementary Table S4). We calculated the proportion of the two groups in each of the hierarchical cluster/sub-cluster of the 4 breast cancer types. One sided *t*-test was also done to compare the differences of average hybridization signals of organisms in these two groups. Nominal *p*-values along with log fold change were calculated. Microbial signatures that were detected with significantly (nominal *p*-value < 0.05) higher average hybridization signal in the deceased cases or in the patients that survived were selected to do box plots for representation of the data. Also differences in the detection of some signatures which were not statistically significant between the different outcomes, but showed some trend were plotted as well. Where, the *p*-value > 0.05, we can only suggest that higher detection of those microbial signatures with either of the outcome, is only seen as a trend.

### PCR validation of pathochip results

PCR primers from the conserved and/or specific regions of the micro-organisms detected by PathoChip screen were used. The PCR amplification reaction mixtures for each reaction contained 200-400 ng of WTA product and 20 pM each of forward and reverse primers (**Table 7**), 300 μM of dNTPs and 2.5U of LongAmp Taq DNA polymerase (NEB). DNA was denatured at 94°C for 3 min, followed by 30 cycles of 94°C for 30 s, different annealing temperature for different set of primers for 30–45 s, and 65°C for 30 s. The PCR conditions for each of the primer sets are mentioned in **Table 7**.

## Results

### Microbial signatures associated with different breast cancer types

**Figure 1:**
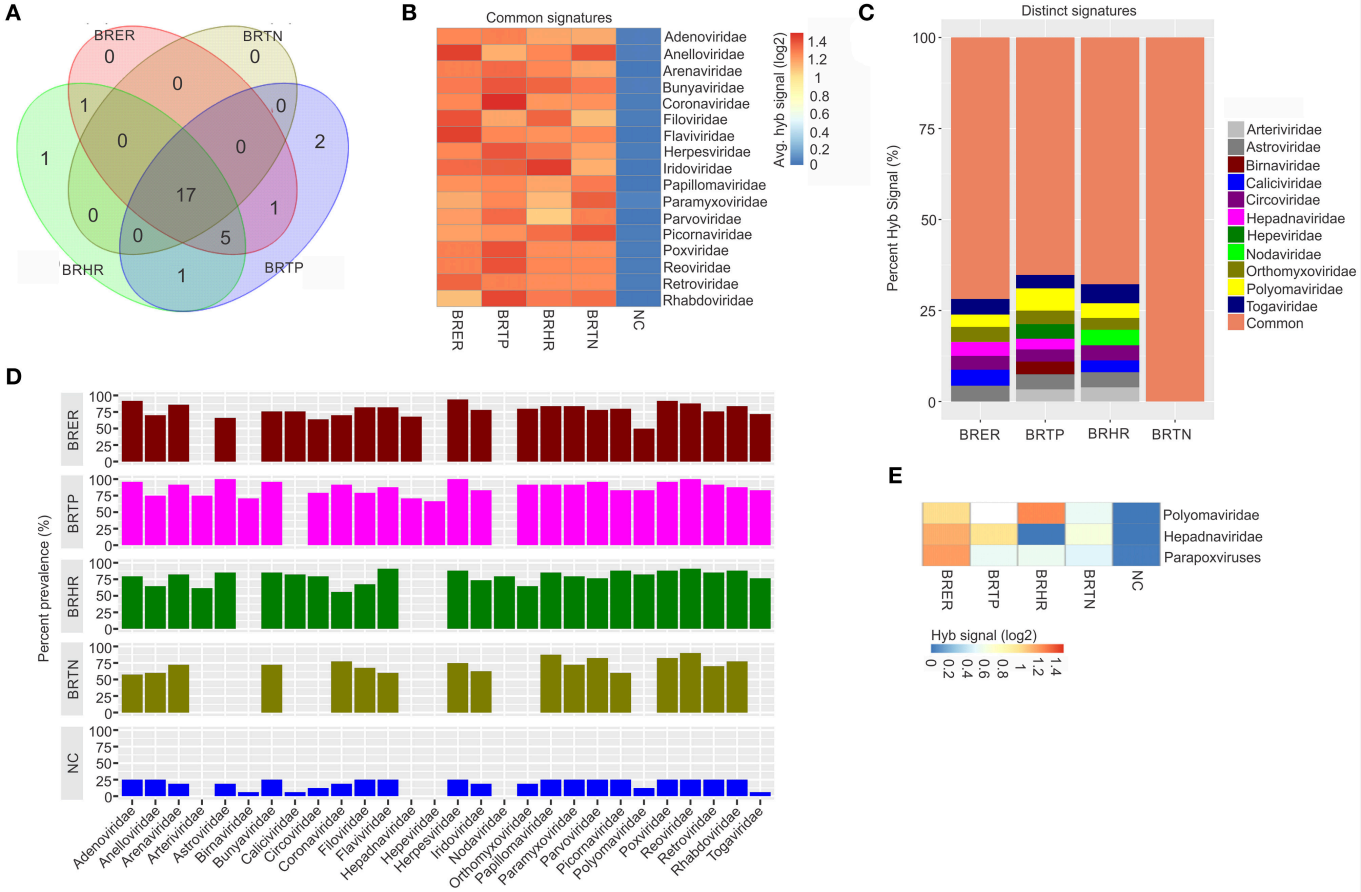
Viral signatures associated with different breast cancer types. Among the breast cancer types, the endocrine receptor (estrogen/progesterone) positives are abbreviated as BRER, human epidermal growth factor receptor 2 positives are abbreviated as BRHR, triple positives (estrogen, progesterone and HER2 receptor positive) are abbreviated as BRTP and the triple negatives (absence of estrogen, progesterone and HER2 receptors) are abbreviated as BRTN. The normal breast control samples obtained from healthy individuals are abbreviated as NC. **(A)** Venn diagram showing the common and unique viral signatures in the 4 types of breast cancers. **(B)** The heat map of common viral signatures in the 4 breast cancer types. **(C)** Relative hybridization signals of viral probes detected in breast cancer types. For example, hybridization signals for Polyomaviridae probes were 4, 6, and 3% of the total hybridization signals detected in BRER, BRTP, and BRHR respectively. **(D)** Prevalence of viral signatures in 4 breast cancer types. Since the hybridization signals for Polyomaviridae, Hepadnaviridae and Parapoxviridae were lower than the cut-off (log2 fold change in hybridization signal >1) in one or more breast cancer types they are depicted as negative in this figure. However, **(E)** shows the heat map of hybridization signals for those viral signatures to be still significantly higher in the cancers when compared to the control.

**Figure 2:**
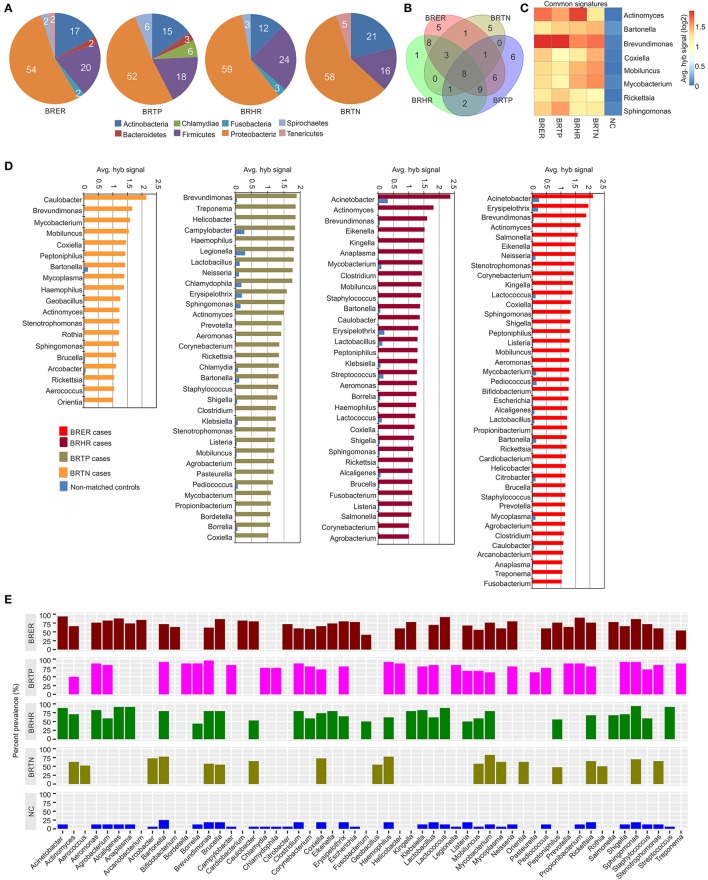
Bacterial signatures associated with different breast cancer types. Among the breast cancer types, the endocrine receptor (estrogen/progesterone) positives are abbreviated as BRER, human epidermal growth factor receptor 2 positives are abbreviated as BRHR, triple positives (estrogen, progesterone and HER2 receptor positive) are abbreviated as BRTP and the triple negatives (absence of estrogen, progesterone and HER2 receptors) are abbreviated as BRTN. The normal breast control samples obtained from healthy individuals are abbreviated as NC. **(A)** Bacterial phyla associated with breast cancer types. **(B)** Venn diagram showing the common and unique bacterial signatures in the 4 types of breast cancers **(C)**. The heat map of common viral signatures in the 4 breast cancer types. **(D)** Hybridization signals of bacterial probes detected in breast cancer types. **(E)** Prevalence of bacterial signatures in 4 breast cancer types.

**Figure 3:**
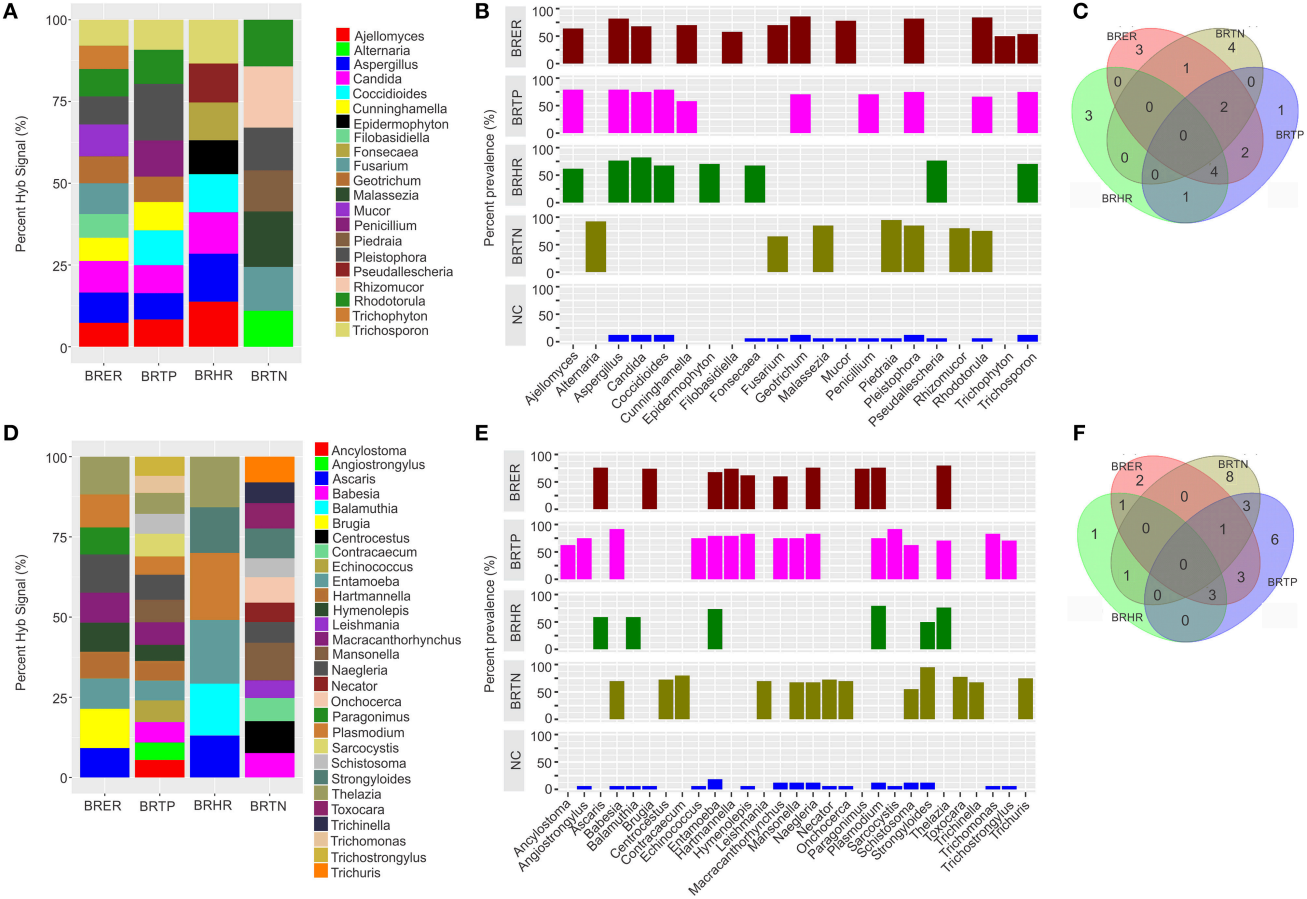
Fungal and parasitic signatures associated with different breast cancer types. Among the breast cancer types, the endocrine receptor (estrogen/progesterone) positives are abbreviated as BRER, human epidermal growth factor receptor 2 positives are abbreviated as BRHR, triple positives (estrogen, progesterone, and HER2 receptor positive) are abbreviated as BRTP and the triple negatives (absence of estrogen, progesterone, and HER2 receptors) are abbreviated as BRTN. The normal breast control samples obtained from healthy individuals are abbreviated as NC. **(A)** Relative hybridization signals of fungal probes detected in breast cancer types. For example hybridization signals for *Ajellomyces* were 7, 8, and 14% of the total hybridization signals detected in BRER, BRTP, and BRHR respectively, and that of *Rhizomucor* is 19% of the hybridization signals detected in BRTN. **(B)** Prevalence of viral signatures in 4 breast cancer types. **(C)** Venn diagram showing the common and unique fungal signatures in the 4 types of breast cancers. **(D)** Relative hybridization signals of parasitic probes detected in breast cancer types. For example hybridization signals for *Plasmodium* were 10, 6, and 21% of the total hybridization signals detected in BRER, BRTP, and BRHR respectively, and that of *Mansonella* is 7 and 12% of the hybridization signals detected in BRTP and BRTN respectively. **(E)** Prevalence of parasitic signatures in 4 breast cancer types. **(F)** Venn diagram showing the common and unique parasitic signatures in the 4 types of breast cancers.

Unique and common microbial signatures associated with different breast cancer types have been listed in Table 1 and are represented in Figures 1A, 2B, 3C,F. To establish the microbial signatures in the cancers we compared the average hybridization signal for each probe in the cancer samples vs. the controls. Those probes that detected significant higher hybridization signals in the cancer samples (*p*-value < 0.05, log2 fold change in hybridization signal > 1), present in atleast 40% of the cancer samples, and ≤25% of the controls were considered in the present study. A stringent cut-off criteria of microbial signature detections only in the cancers and not (0% prevalence) in the controls lead mostly to detect less number of probes for a particular signature for some signatures, not for all, but not that the majority signatures detected with our accepted cut-off was lost (Supplementary Figures S2a–d).

**Table 1:** Unique and common microbial signatures in 4 breast cancer types; the endocrine receptor positives (BRER), human epidermal growth factor receptor 2 positives (BRHR), triple positives (BRTP) and the triple negatives (BRTN).

**Cancer types**	**Viral signatures**	**Bacterial signatures**	**Fungal signatures**	**Parasitic signatures**
BRER		Arcanobacterium, Bifidobacterium, Cardiobacterium, Citrobacter, Escherichia	Filobasidilla, Mucor, Trichophyton	Brugia, Paragonimus
BRHR	Nodaviridae	Streptococcus	Epidermophyton, Fonsecaea, Pseudallescheria	Balamuthia
BRTP	Birnaviridae, Hepeviridae	Bordetella, Campylobacter, Chlamydia, Chlamydophila, Legionella, Pasteurella	Penicillium	Ancylostoma, Angiostrongylus, Echinococcus, Sarcocystis, Trichomonas, Trichostrongylus
BRTN		Aerococcus, Arcobacter, Geobacillus, Orientia, Rothia	Alternaria, Malassezia, Piedraia, Rhizomucor	Centrocestus, Contracaecum, Leishmania, Necator, Onchocerca, Toxocara, Trichinella, Trichuris
BRTN+BRER		Mycoplasma		
BRTN+BRTP				Babesia, Mansonella, Schistosoma
BRER+BRTN			Fusarium	
BRER+BRHR	Caliciviridae	Acinetobacter, Alcaligenes, Anaplasma, Eikenella, Fusobacterium, Kingella, Lactococcus, Salmonella		Ascaris
BRHR+BRTP	Arteriviridae	Borrelia, Klebsiella	Coccidioides	
BRHR+BRTN				Strongyloides
BRER+BRTP	Hepadnaviridae	Helicobacter, Neisseria, Pediococcus, Prevotella, Propionibacterium, Treponema	Cunninghamella, Geotrichum	Hartmannella, Hymenolepis, Macracanthorhynchus
BRTN+BRER+BRTP		Stenotrophomonas	Pleistophora, Rhodotorula	Naegleria
BRTN+BRER+BRHR		Brucella, Caulobacter, Peptoniphilus		
BRHR+BRTN+BRTP		Haemophilus		
BRHR+BRTP+BRER	Astroviridae, Circoviridae, Orthomyxoviridae, Polyomaviridae, Togaviridae	Agrobacterium, Clostridium, Corynebacterium, Erysipelothrix, Lactobacillus, Listeria, Shigella, Staphylococcus	Ajellomyces, Aspergillus, Candida, Trichosporon	Entamoeba, Plasmodium, Thelazia
BRER+BRHR+BRTP +BRTN	Adenoviridae, Anelloviridae, Arenaviridae, Bunyaviridae, Coronaviridae, Filoviridae, Flaviviridae, Herpesviridae, Iridoviridae, Papillomaviridae, Paramyxoviridae, Parvoviridae, Picornaviridae, Poxviridae, Reoviridae, Retroviridae, Rhabdoviridae	Actinomyces, Bartonella, Brevundimonas, Coxiella, Mobiluncus, Mycobacterium, Rickettsia, Sphingomonas		

We further averaged the hybridization signals of all the significant probes for each microbial genera and viral families, shown in the Figures 1–3. Supplementary Table S1 shows the average hybridization signals of the probes of microorganisms significantly detected in the cancers vs. the controls, with respective adjusted *p*-values with multiple corrections. Supplementary Table S2 shows the proportion of probes that were detected significantly in each of the breast cancer types vs. the controls. Supplementary Figure S1 shows the average fold change in hybridization signal intensity for the significantly detected probes of each of the signatures detected in the different breast cancer types over their respective signals in the control breast samples. Additionally, we calculated the percent prevalence of the significant microbial signatures in the cancer samples, which indicate how prevalent a significant virus or microorganism signature is in the cancer samples regardless of the hybridization intensity.

### Viral signatures associated with different breast cancer types

Significant hybridization (described above), at levels above the controls, was detected for 28 viral families among the four breast cancer types (Figures 1A,D). Of these, 17 viral families were detected with significantly higher hybridization signals in greater than 50% of the samples representing all 4 breast cancer types, as compared to the controls (Figures 1B,D). They include signatures of Adenoviridae, Anelloviridae, Arenaviridae, Bunyaviridae, Coronaviridae, Filoviridae, Flaviviridae, Herpesviridae, Iridoviridae, Papillomaviridae, Paramyxoviridae, Parvoviridae, Picornaviridae, Poxviridae, Reoviridae, Retroviridae, and Rhabdoviridae (Figure 1B). Importantly, in examining the percent hybridization signal (Figure 1C) and percent prevalence (Figure 1D) we find that there were a number of viral families significantly detected only in a subset of breast cancer types. Specifically, the signatures for Birnaviridae and Hepeviridae were only detected in BRTP; and Nodaviridae only in BRHR (Figures 1C,D). Further examination of the percent prevalence (Figure 1D), shows that BRTN samples show low or no prevalence of Arteriviridae, Astroviridae, Birnaviridae, Caliciviridae, Circoviridae, Hepadnaviridae, Nodaviridae, Orthomyxoviridae, Polyomaviridae, and Togaviridae; BRHR samples show low or no prevalence of Birnaviridae, Hepadnaviridae, and Hepeviridae; BRTP samples show low or no prevalence of Caliciviridae and Nodaviridae; and BRER samples show low or no prevalence of Arteriviridae, Birnaviridae, Hepeviridae, and Nodaviridae.

Hybridization signal intensity offers an additional way to compare the data. Here we noted marked differences for specific viral families between the different breast cancer types. For example, probes for polyomaviridae were detected with the highest hybridization signal in the BRHRs, followed by BRERs and BRTPs (Figure 1E). Polyomaviridae were detected in the BRTNs compared to the controls; however, at a lower hybridization signal (log2 fold change in hybridization signal = 0.4–1; Figure 1E) which is below the cut-off to consider the signal positive, thus polyomaviridae are not shown to be present in the BRTNs in Figure 1C or Figure 1D. Similarly, probes of Hepadnaviridae were significantly detected with low hybridization signal in the BRTNs (Figure 1E), while detected with higher hybridization signal intensity (log2 fold change in hybridization signal >1) in the BRERs and BRTPs (Figure 1E).

Signatures of Herpesviridae, Adenoviridae and Poxviridae were detected in >90% of the BRER samples screened (Figure 1D), while the highest hybridization signal was detected for Anelloviridae and Flaviviridae (Figure 1B). Signatures of Astroviridae, Herpesviridae, Reoviridae were detected in all of the BRTP samples tested (Figure 1D), with the highest hybridization signal detected for Polyomaviridae signatures (Figure 1C). For BRHR samples, signatures of Reoviridae and Flaviviridae were detected in >90% of the samples screened (Figure 1D), with signatures of Togaviridae showing the highest hybridization signal (Figure 1C). Among the BRTN samples, we detected signatures of Reoviridae in 90% of the samples screened (Figure 1D), with signatures of Picornaviridae and Anelloviridae with the highest hybridization signal (Figure 1B).

Probes of Poxviridae family were detected significantly in >80% of all the breast cancer types analyzed. Interestingly, probes of Parapoxviridae were detected significantly with high hybridization signal intensity in BRER cancers vs. the controls (Figure 1E). Probes of Parapoxviridae were also detected significantly in the other 3 types of breast cancers compared to the controls, but showed much lower hybridization signal intensity for those probes (log2 fold change in hybridization signal ~0.5) (Figure 1E).

The data show that the cancer samples as a whole have a robust viral signature. However, there are significant and defining differences between the four types with BRTN having the least complex viral signature.

In the healthy control breast tissues, signatures of the viral families Arteriviridae, Hepadnaviridae, Hepeviridae, and Nodaviridae were not detected which were detected in one or more of the cancer types (Figure 1D).

### Bacterial signatures associated with different breast cancer types

Figures 2A–E shows the analysis of bacterial signatures in the 4 breast cancer types. Significant hybridization, above the levels of the controls, was detected for 56 bacterial genera; the majority (50–60%) was proteobacteria, the major group of gram negative bacteria. These phyla partitioned into bacterial signatures unique to each cancer types, as well as signatures that were common to multiple breast cancer types (Table 1, Figures 2B–D). Significant hybridization signals common to all 4 breast cancer types were detected for *Actinomyces, Bartonella, Brevundimonas, Coxiella, Mobiluncus, Mycobacterium, Rickettsia*, and *Sphingomonas* (Figures 2B,C).

The marked diversity in bacterial signatures between the breast cancer types are shown in Figure 2B. We identified distinct bacterial signatures uniquely associated with each type of breast cancer analyzed. In this regard BRTN had the least complex bacterial signature, while BRER is the most complex (Figures 2D,E). Signals for *Arcanobacterium, Bifidobacterium, Cardiobacterium, Citrobacter, Escherichia* were significantly detected in the BRER samples compared to the controls, while those of *Bordetella, Campylobacter, Chlamydia, Chlamydophila, Legionella*, and *Pasteurella* were significantly associated with the BRTPs. Signals for *Streptococcus* were detected significantly in the BRHRs, whereas, *Aerococcus, Arcobacter, Geobacillus, Orientia*, and *Rothia* were found associated with the BRTNs.

Hybridization signal intensity again provides an additional view of the complexity of the bacterial community and its diversity among the different breast cancers (Figures 2C,D). Signals for *Brevundimonas* were detected with higher average hybridization signals in the endocrine receptor positive BRER and BRTP compared to the endocrine receptor negative BRHR and BRTN (Figures 2C,D). Hybridization signals of *Mobiluncus* and *Mycobacterium* were predominantly detected in the endocrine receptor negative samples.

Bacterial signatures of *Actinomyces* were detected in all 4 cancer types; however their hybridization signal intensity was markedly lower in the BRTN samples (Figure 2C). Similarly, *Bartonella* was significantly detected in all cancer types, but its hybridization signal intensity was markedly lower in the BRER samples compared to the others (Figure 2C). The bacterial probes detected with the highest hybridization signals were those for *Acinetobacter* in BRER and BRHR samples, *Brevundimonas* in BRTP samples and *Caulobacter* in BRTN samples (Figure 2D). As in the case of the viruses our data show that the cancer samples have a robust bacterial signature with significant and defining differences between the four breast cancer types. The healthy control samples did not have some of the bacterial signatures that were detected in one or more of the cancer types, namely, *Actinomyces, Aerococcus, Arcanobacterium, Bifidobacterium, Bordetella, Cardiobacterium, Corynebacterium, Eikenella, Fusobacterium, Geobacillus, Helicobacter, Kingella, Orientia, Pasteurella, Peptinophilus, Prevotella, Rothia, Salmonella*, and *Treponema* (Figure 2E).

### Fungal signatures associated with different breast cancer types

Significant hybridization, above the levels of the controls, was detected for 21 different genera of fungi among the 4 types of breast cancer (Figures 3A,B). Interestingly, none of these families were detected in all four cancer types (Figures 3B,C). In fact the fungi signatures for each type of breast cancer were relatively unique; only 7 fungal families (*Aspergillus, Candida, Coccidioides, Cunninghamella, Geotrichum, Pleistophora*, and *Rhodotorula*) were detected in more than one type of breast cancer. The receptor positive cancer samples (BRER and BRTPs) showed much more complex fungal diversity than the BRTN samples (Figures 3A,B). Table 1 and Figure 3C show the unique fungal signatures associated with different breast cancer types. Fungal signatures of *Filobasidiella, Mucor*, and *Trichophyton* were found to be significantly associated with BRER samples, *Penicillium* with BRTP samples, *Epidermophyton, Fonsecaea, Pseudallescheria* with BRHR samples and *Alternaria, Malassezia, Piedraia*, and *Rhizomucor* with BRTN samples. Fungal signatures of *Ajellomyces, Alternaria, Cunninghamella, Epidermophyton, Filobasidiella, Rhizomucor*, and *Trichophyton* detected in one or more breast cancer types were not detected in the healthy controls (Figure 3B).

### Parasitic signatures associated with different breast cancer types

Significant hybridization, above the levels of the controls, was detected for 29 different genera of parasites among the 4 types of breast cancer (Figures 3D,E). As in the case of the fungi, no single genus of parasite was significantly detected in all four breast cancer types (Figures 3E,F). Each cancer showed a relatively distinct parasitic signature pattern, with BRHR showing the least diverse signatures. Table 1 and Figure 3F shows the unique and common parasitic signatures among the different breast cancer types.

Analysis of hybridization signal intensity in Figure 3D shows that *Plasmodium* was detected with the highest hybridization signal in the BRHR samples and also detected in the BRER samples and BRTP samples but not in BRTN samples. In BRTN the highest hybridization signal intensity was detected for the probes of *Mansonella* followed by *Centrocestus*, whereas *Strongyloides* was detected in almost all of the BRTN samples. *Naegleria* was detected with the highest hybridization signal intensity in BRTP (Figure 3D) while *Sarcocystis* and *Babesia* were detected in 92% of BRTP samples (Figure 3E). Among the BRER samples, *Brugia* showed the highest hybridization signal intensity (Figure 3D), while *Thelazia* showed the highest prevalence (Figure 3E). Signatures of *Brugia* and *Paragonimus* were only detected in BRER samples (Table 1, Figures 3D,E). *Ancylostoma, Angiostrongylus, Echinococcus, Sarcocystis, Trichomonas, Trichostrongylus* were found uniquely associated with BRTP samples (Table 1, Figures 3D–F). *Balamuthia* signatures were associated significantly with BRHR samples, and that of *Centrocestus, Contracaecum, Leishmania, Necator, Onchocerca, Toxocara, Trichinella*, and *Trichuris* were detected significantly only with BRTN samples (Table 1, Figures 3D–F). Signatures of *Ancylostoma, Ascaris, Centrocestus, Contracaecum, Hartmanella, Leishmania, Paragonimus, Thelazia, Toxocara, Trichinella, Trichuris* detected in one or more cancer types were not detected in the healthy controls (Figure 3E).

### Hierarchical clustering of the breast cancer samples based on the detection of microbial signatures

**Figure 4:**
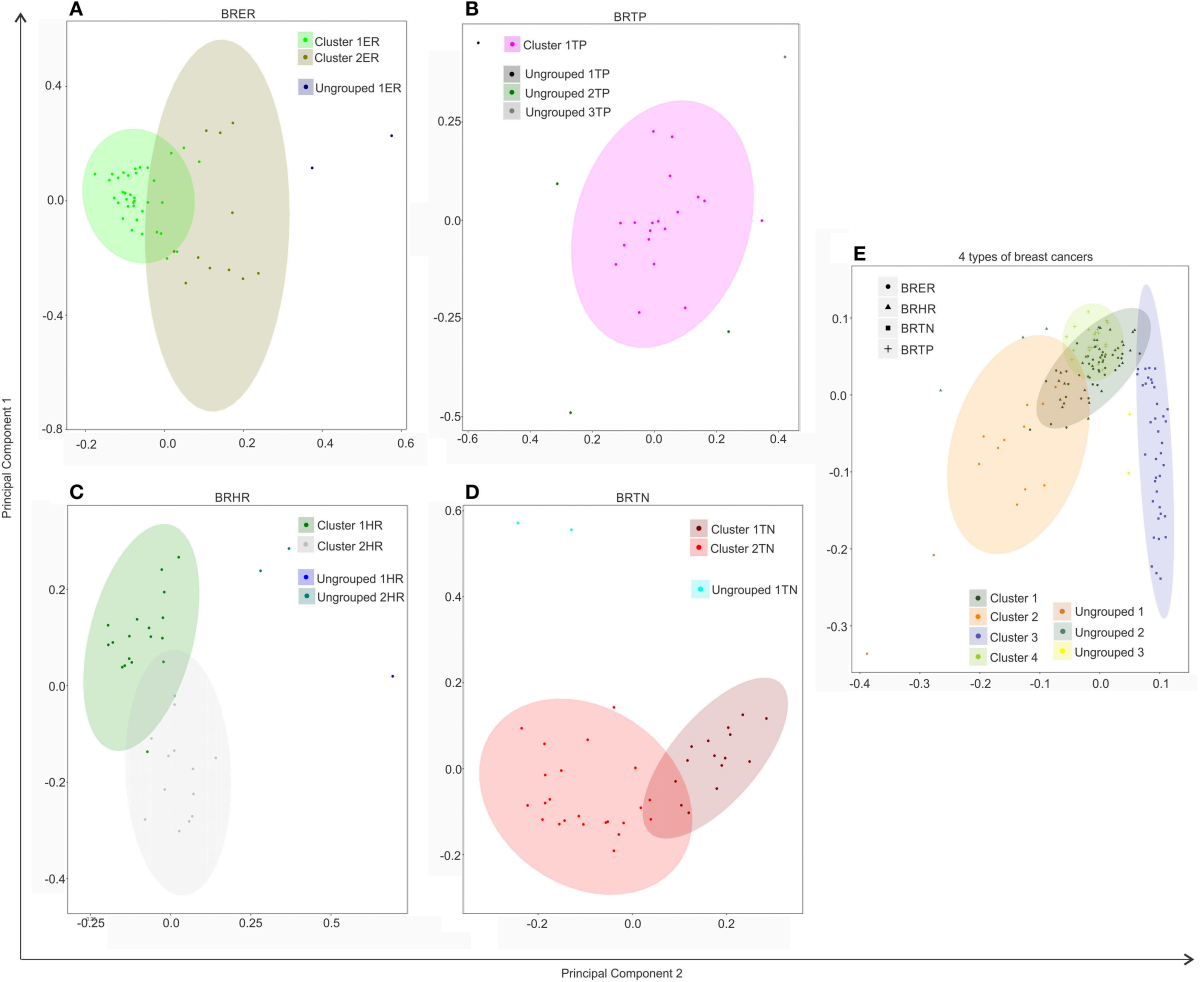
Hierarchical clustering of BRER **(A)**, BRTP **(B)**, BRHR **(C)**, BRTN **(D)**, and all 4 breast cancer types **(E)** based on their microbial signature detection pattern. The endocrine receptor (estrogen/progesterone) positive breast cancers are abbreviated as BRER, human epidermal growth factor receptor 2 positive breast cancers are abbreviated as BRHR, triple positive (estrogen, progesterone, and HER2 receptor positive) breast cancers are abbreviated as BRTP and the triple negative (absence of estrogen, progesterone, and HER2 receptors) breast cancers are abbreviated as BRTN.

Using the hierarchical clustering analysis based on the detection of microbial signatures associated with the 4 breast cancer types we determine if the breast cancer types fell into any unique and identifiable clusters. While this analysis identified distinct clusters in each of the breast cancer types based on the detection of their microbial signature patterns (Figures 4A–D), it also defined the distinct microbial signature pattern found in BRTNs and BRTPs whereas, BRER and BRHR shared similar microbial signatures (Figure 4E).

Individually, the different BC types fell into distinct microbial signature clusters. BRER samples fell into 2 distinct clusters 1ER and 2ER, along with 2 ungrouped samples (ungrouped 1ER) (Figure 4A). Samples grouped in Cluster 1ER and 2ER differed significantly based on the higher detection of mostly bacterial and viral and certain fungal and parasitic signatures in the samples of Cluster 2ER (Table 2). The ungrouped BRER samples (ungrouped 1ER) were significantly different from clusters 1ER and 2ER (Table 2).

**Table 2:** Significant differences in microbial signatures between the hierarchical clusters of the endocrine receptor positive breast cancers (BRER).

**Organism**	**T statistic**	***P*-value**	**LogFC**
**BRER CLUSTER 1ER VS. CLUSTER 2ER IN FIGURE 4A**			
Propionibacterium	−7.211	7.39E−07	−1.557
Arcanobacterium	−7.194	2.25E−06	−1.616
Brucella	−6.512	2.76E−05	−1.613
Adenoviridae	−5.232	9.03E−05	−0.901
Brevundimonas	−5.243	0.000166	−3.275
Caulobacter	−5.296	0.0002	−1.858
Pleistophora	−4.763	0.000346	−1.617
Eikenella	−4.538	0.000435	−2.023
Poxviridae	−4.644	0.000443	−0.823
Filoviridae	−3.886	0.00056	−0.906
Alcaligenes	−4.288	0.000854	−1.525
Kingella	−4.275	0.000879	−1.954
Reoviridae	−4.019	0.001078	−0.733
Flaviviridae	−4.083	0.001118	−1.037
Retroviridae	−3.926	0.001159	−1.087
Erysipelothrix	−3.928	0.00116	−1.730
Herpesviridae	−4.253	0.001288	−1.083
Orthomyxoviridae	−4.033	0.001308	−1.030
Papillomaviridae	−4.112	0.001326	−1.076
Shigella	−3.685	0.001537	−1.190
Astroviridae	−3.349	0.001945	−0.795
Citrobacter	−3.552	0.002306	−1.063
Macracanthorhynchus	−3.680	0.002931	−1.784
Rickettsia	−3.409	0.004689	−1.169
Coxiella	−3.490	0.004739	−2.031
Trichophyton	−3.566	0.00481	−2.754
Acinetobacter	−3.281	0.004871	−1.544
Escherichia	−3.366	0.005736	−1.493
Mycoplasma	−3.250	0.006671	−1.627
Staphylococcus	−3.098	0.007102	−0.751
Paramyxoviridae	−3.059	0.007333	−0.621
Aeromonas	−3.024	0.007923	−0.984
Pediococcus	−3.129	0.008223	−1.645
Mucor	−3.220	0.008331	−2.103
Brugia	−3.065	0.009069	−1.711
Paragonimus	−2.766	0.014456	−1.021
Polyomaviridae	−2.750	0.015486	−1.258
Rhabdoviridae	−2.825	0.016266	−0.913
Stenotrophomonas	−2.808	0.016458	−1.617
Parvoviridae	−2.594	0.017333	−0.465
Rhodotorula	−2.723	0.018918	−0.915
Cardiobacterium	−2.726	0.020259	−1.386
Prevotella	−2.521	0.020957	−0.911
Treponema	−2.660	0.022602	−1.820
Circoviridae	−2.587	0.02349	−1.135
Bunyaviridae	−2.544	0.025934	−0.772
Hymenolepis	−2.547	0.026229	−1.566
Hartmannella	−2.452	0.027852	−0.837
Plasmodium	−2.334	0.029775	−0.646
Sphingomonas	−2.440	0.033178	−1.495
Corynebacterium	−2.314	0.036828	−1.463
Lactobacillus	−2.284	0.040908	−0.887
Anelloviridae	−2.246	0.041582	−0.867
Aspergillus	−2.232	0.046187	−1.222
**BRER CLUSTER 1ER VS. UNGROUPED 1ER IN FIGURE 4A**			
Pleistophora	−28.365	4.78E−13	−4.105
Brugia	−7.866	1.87E−08	−1.653
Escherichia	−30.534	6.54E−08	−4.933
Neisseria	−6.310	4.77E−07	−1.466
Retroviridae	−13.206	0.000123	−2.407
Coxiella	−11.147	0.000156	−2.297
Togaviridae	−4.362	0.000578	−0.742
Papillomaviridae	−9.237	0.011952	−1.470
Citrobacter	−12.427	0.011967	−3.997
Brucella	−12.872	0.013747	−2.140
Corynebacterium	−7.594	0.015709	−3.346
Fusobacterium	−8.502	0.040745	−5.272
Rickettsia	−2.767	0.045012	−0.457
**BRER CLUSTER 2ER VS. UNGROUPED 1ER IN FIGURE 4A**			
Retroviridae	−4.822	0.00084	−1.320
Citrobacter	−7.684	0.003969	−2.934
Corynebacterium	−2.730	0.027196	−1.883
Escherichia	−7.864	8.52E−06	−3.439
Fusobacterium	−6.841	0.017391	−4.806
Neisseria	−2.315	0.042091	−1.067
Pleistophora	−7.693	1.07E−05	−2.489

Majority of the BRTP samples had similar microbial detections and grouped together into 1 major cluster (cluster 1TP), while few samples remained ungrouped (Figure 4B).

The BRHR samples formed 2 major clusters (cluster 1HR and cluster 2HR) (Figure 4C), and they differed from each other in having higher detection of certain bacterial and viral signatures in cluster 2HR compared to samples in cluster 1HR (Table 3). Bacterial signatures of *Kingella, Brevundimonas, Eikenella, Bartonella, Acinetobacter, Nodaviridae, Actinomyces, Aeromonas, Mobiluncus, Fusobacterium, Alcaligenes, Brucella*, and *Staphylococcus*; viral signatures of Orthomyxoviridae, Parvoviridae, Papillomaviridae, Nodaviridae, and Astroviridae and fungal signatures of *Aspergillus* showed significant higher detection in cluster 2HR. The 3 BRHRs that could not be grouped (ungrouped 1HR and 2HR) showed higher detection of certain microbial signatures listed in Table 3 compared to the clustered BRHR samples; in particular, included the parasitic signature of *Entamoeba* and bacterial signatures of *Listeria* and *Corynebacterium*.

**Table 3:** Significant differences in microbial signatures between the hierarchical clusters of the human epidermal growth factor receptor 2 positive breast cancers (BRHR).

**Organism**	**T statistic**	***P*-value**	**LogFC**
**BRHR CLUSTER 1HR VS. CLUSTER 2HR IN FIGURE 4C**			
Kingella	−8.239	8.7E−09	−2.185
Brevundimonas	−9.967	2.3E−08	−2.411
Eikenella	−6.587	4.2E−07	−2.008
Bartonella	−7.715	5.8E−07	−1.759
Acinetobacter	−5.766	9.3E−06	−2.858
Nodaviridae	−5.598	1.4E−05	−2.066
Actinomyces	−5.389	1.8E−05	−2.445
Aeromonas	−6.501	1.9E−05	−2.143
Mobiluncus	−4.808	9.1E−05	−2.027
Fusobacterium	−4.200	0.00059	−1.741
Alcaligenes	−4.352	0.00065	−1.164
Brucella	−3.442	0.00319	−0.891
Orthomyxoviridae	3.186	0.00422	1.180
Parvoviridae	−2.993	0.00666	−0.516
Aspergillus	−2.517	0.02244	−1.169
Papillomaviridae	−2.427	0.02385	−0.449
Staphylococcus	−2.073	0.04818	−1.147
Astroviridae	−2.128	0.04831	−0.642
**BRHR CLUSTER 1HR VS. UNGROUPED(1HR**+**2HR), IN FIGURE 4C**			
Lactobacillus	−8.722	5E-07	−3.149
Paramyxoviridae	−8.701	7E-07	−1.295
Entamoeba	−14.273	4E-05	−6.228
Anaplasma	−9.105	2E-04	−1.783
Astroviridae	−7.437	0.003	−1.825
Staphylococcus	−3.735	0.004	−1.758
Brucella	−6.840	0.011	−2.152
Mobiluncus	−5.976	0.011	−3.399
Rhabdoviridae	−6.586	0.013	−2.423
Klebsiella	−3.389	0.017	−2.348
Actinomyces	−3.952	0.02	−2.016
Candida	−4.990	0.025	−2.924
Flaviviridae	−5.242	0.026	−2.677
Caliciviridae	−3.043	0.027	−1.220
Ascaris	−3.655	0.03	−2.466
Arenaviridae	−4.007	0.035	−2.163
Corynebacterium	−4.706	0.038	−4.514
Lactococcus	−2.652	0.04	−0.769
Bartonella	−4.347	0.042	−1.957
Listeria	−4.206	0.043	−3.924
Thelazia	−3.931	0.045	−3.126
Papillomaviridae	−3.901	0.048	−1.585
Anelloviridae	−2.520	0.048	−1.859
Poxviridae	−4.155	0.049	−2.570
**BRHR CLUSTER 2HR VS. UNGROUPED(1HR+2HR) IN FIGURE 4C**			
Entamoeba	−15.712	0.0001	−6.501
Paramyxoviridae	−4.736	0.0003	−1.116
Lactobacillus	−4.561	0.0005	−2.908
Anaplasma	−4.400	0.0007	−1.343
Nodaviridae	4.850	0.0032	2.267
Astroviridae	−3.418	0.0068	−1.183
Rhabdoviridae	−4.586	0.008	−1.980
Candida	−5.384	0.0193	−3.190
Klebsiella	−4.734	0.0197	−2.750
Brucella	−3.320	0.0209	−1.261
Flaviviridae	−4.883	0.021	−2.601
Listeria	−4.088	0.026	−4.122
Arenaviridae	−3.471	0.0344	−1.991
Ascaris	−3.922	0.0368	−2.499
Anelloviridae	−3.885	0.0375	−2.349
Corynebacterium	−4.125	0.0408	−4.068

The BRTN samples formed two distinct clusters (cluster 1TN and 2TN) with 2 samples that did not cluster into distinct group (ungrouped 1TN) (Figure 4D). Cluster 1TN differed from Cluster 2TN in having higher detection of bacterial probes of *Caulobacter, Brevundimonas, Peptoniphilus, Rothia, Geobacillus, Aerococcus, Mobiluncus, Actinomyces, Bartonella*, fungal probes of *Malassezia, Piedraia, Rhodotorula, Rhizomucor* and parasitic signatures of *Leishmania, Toxocara, Contracaecum, Centrocestus, Trichuris, Strongyloides* (Table 4). Whereas, samples in Cluster 2TN had significant higher hybridization signal intensity for viral signatures of Poxviridae, Paramyxoviridae, Reoviridae, Parvoviridae, Arenaviridae, bacterial signatures of *Sphingomonas, Brucella, Orientia, Stenotrophomonas*, fungal signatures of *Pleistophora* and parasitic signatures of *Trichinella*. The ungrouped samples differed from the grouped samples in having significantly higher detection of certain viral probes of Anelloviridae, Retroviridae, Poxviridae, and Arenaviridae compared to Cluster 1TN and Cluster 2TN samples (Table 4).

**Table 4:** Significant differences in microbial signatures between the hierarchical clusters of the triple negative breast cancers (BRTN).

**Organism**	**T statistic**	***P*-value**	**LogFC**
**BRTN CLUSTER 1TN VS CLUSTER 2TN IN FIGURE 4D**			
Caulobacter	14.725	5E-16	4.006
Brevundimonas	16.431	5E-15	3.496
Peptoniphilus	12.519	9E-11	3.326
Rothia	10.847	4E-10	2.647
Geobacillus	11.926	3E-09	2.917
Aerococcus	12.298	3E-09	2.544
Mobiluncus	8.106	1E-08	2.793
Leishmania	6.619	2E-07	1.256
Actinomyces	6.417	2E-07	1.837
Malassezia	6.015	1E-06	1.598
Toxocara	5.734	4E-06	1.387
Contracaecum	5.814	4E-06	1.176
Piedraia	5.368	6E-06	0.815
Rhodotorula	5.298	6E-06	1.402
Centrocestus	5.325	2E-05	1.720
Rhizomucor	5.023	3E-05	1.413
Trichuris	4.913	3E-05	1.267
Strongyloides	4.814	4E-05	1.110
Bartonella	4.524	2E-04	1.901
Poxviridae	−3.959	3E-04	−0.504
Paramyxoviridae	−3.773	6E-04	−1.022
Sphingomonas	−3.686	9E-04	−1.264
Pleistophora	3.640	0.001	1.097
Reoviridae	−2.987	0.005	−0.539
Trichinella	2.916	0.006	1.062
Arenaviridae	−2.845	0.008	−0.713
Brucella	−2.748	0.01	−1.098
Orientia	−2.521	0.018	−0.942
Parvoviridae	−2.294	0.028	−0.701
Stenotrophomonas	−2.231	0.032	−0.533
**BRTN CLUSTER 1TN VS CLUSTER 1TN IN FIGURE 4D**			
Anelloviridae	−18.960	4E-05	−8.958
Retroviridae	−20.048	6E-11	−6.108
Poxviridae	−25.133	1E-13	−1.989
Arenaviridae	−10.652	4E-08	−1.201
Iridoviridae	3.061	0.008	0.972
Mycoplasma	2.912	0.011	1.102
Trichinella	4.806	3E-04	1.146
Rickettsia	2.916	0.011	1.156
Adenoviridae	2.848	0.013	1.178
Filoviridae	3.956	0.001	1.226
Actinomyces	6.995	4E-05	1.605
Babesia	7.967	1E-06	1.648
Aerococcus	6.799	0.014	2.342
Toxocara	28.244	1E-13	2.495
Rothia	13.228	3E-09	2.874
Centrocestus	13.652	0.036	2.912
Peptoniphilus	14.486	8E-10	3.518
**BRTN CLUSTER 2TN VS. UNGROUPED 1TN IN FIGURE 4D**			
Anelloviridae	−23.294	0.0007	−9.497
Retroviridae	−18.299	2E-12	−5.681
Poxviridae	−14.532	5E-13	−1.485
Arenaviridae	−2.111	0.0459	−0.488
Rickettsia	3.576	0.0017	1.064
Filoviridae	3.714	0.0012	1.100
Toxocara	4.920	6E-05	1.108
Centrocestus	3.124	0.0122	1.192
Adenoviridae	2.834	0.0097	1.249
Iridoviridae	4.805	8E-05	1.341
Babesia	5.584	1E-05	1.556
Mycoplasma	4.207	0.0004	1.701

Figure 4E shows the comparison of the microbial signatures from all four breast cancer types together in the clustering analysis. The data show that the different breast cancers grouped into 4 major clusters plus a few ungrouped BRER (2 samples), BRHR (3 samples), and BRTN (2 samples) samples (ungrouped 1, 2, and 3 respectively). Most of the BRTNs were very distinct in their microbial signature pattern association, and they clustered together (cluster 3). Similarly all the BRTPs screened clustered together to form a distinct cluster 4. Conversely, most of the BRER samples shared a similar microbial signature pattern with all of the BRHR samples forming the distinct cluster 1, while the remaining 11 BRER samples formed cluster 2. The BRERs in cluster 2 differed from those in Cluster 1 in having significant higher hybridization signals for certain bacterial signatures like *Brevundimonas, Sphingomonas, Erysipelothrix, Mycoplasma, Brucella, Prevotella, Arcanobacterium, Staphylococcus, Rickettsia, Propionibacterium, Lactobacillus, Shigella*, viral signatures of Polyomaviridae, Circoviridae, Herpesviridae, Papillomaviridae, Retroviridae, Orthomyxoviridae, Flaviviridae, Iridoviridae, Poxviridae, Reoviridae, fungal signatures of *Trichophyton, Mucor, Rhodotorula, Geotrichum, Pleistophora* and parasitic signatures of *Paragonimus, Macracanthorhynchus, Hartmannella* (Table 5).

**Table 5:** Significant differences in microbial signatures between the hierarchical clusters of the endocrine receptor positive breast cancers of cluster 1 vs. 2 in Figure 4E.

**Organism**	**T statistic**	***P*-value**	**LogFC**	**Types**
Trichophyton	−4.156	0.002	−3.067	Fungal
Mucor	−4.269	0.001	−2.400	Fungal
Brevundimonas	−2.357	0.033	−1.966	Bacterial
Sphingomonas	−3.025	0.012	−1.781	Bacterial
Erysipelothrix	−2.685	0.018	−1.725	Bacterial
Mycoplasma	−3.094	0.009	−1.598	Bacterial
Polyomaviridae	−4.103	7E-04	−1.511	Viral
Paragonimus	−3.974	0.001	−1.448	Parasitic
Macracanthorhynchus	−3.362	0.004	−1.437	Parasitic
Brucella	−4.795	3E-04	−1.419	Bacterial
Circoviridae	−2.985	0.011	−1.292	Viral
Prevotella	−2.306	0.04	−1.291	Bacterial
Hartmannella	−3.531	0.003	−1.183	Parasitic
Rhodotorula	−2.323	0.04	−1.116	Fungal
Herpesviridae	−2.669	0.022	−1.088	Viral
Geotrichum	−2.352	0.034	−1.015	Fungal
Arcanobacterium	−2.949	0.011	−1.013	Bacterial
Pleistophora	−2.163	0.047	−0.989	Fungal
Papillomaviridae	−2.913	0.013	−0.917	Viral
Staphylococcus	−3.037	0.009	−0.909	Bacterial
Retroviridae	−2.452	0.027	−0.876	Viral
Orthomyxoviridae	−3.343	0.005	−0.863	Viral
Rickettsia	−2.321	0.037	−0.852	Bacterial
Flaviviridae	−2.814	0.014	−0.851	Viral
Propionibacterium	−2.354	0.033	−0.834	Bacterial
Iridoviridae	−2.539	0.022	−0.830	Viral
Poxviridae	−2.732	0.019	−0.787	Viral
Lactobacillus	−2.271	0.04	−0.781	Bacterial
Shigella	−2.171	0.042	−0.735	Bacterial
Reoviridae	−2.579	0.023	−0.655	Viral

Thus, we identified specific microbial signature patterns associated with different breast cancer types. It will be interesting to see if such distinct microbial signature pattern associated with different breast cancer types, correlate to differences in pathogenesis and clinical outcome.

### Association of microbial signatures with clinical outcomes in the four breast cancer types

The samples we used in this study were de-identified samples. Thus due to HIPPA regulations we were able to procure only limited sub-set of data from the Tumor Registry. This included outcome, specifically whether the patient was alive or dead since diagnosis and treatment; the cause of death and length of survival were not available. These data provide only indications of trends which will have to be statistically verified in future studies using samples with associated clinical data.

**Figure 5:**
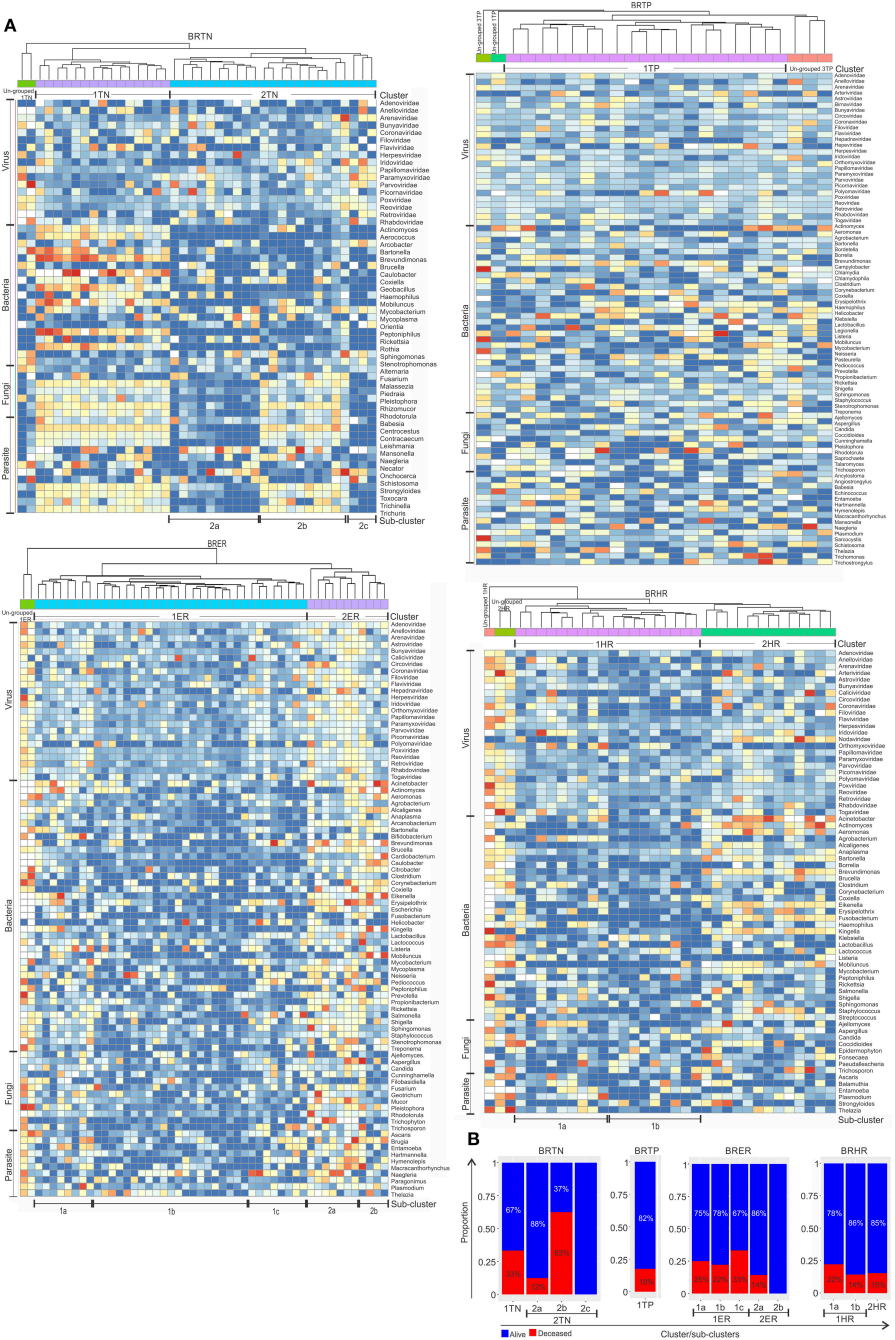
Heat map of hierarchical clustering of the 4 types of breast cancers **(A)**, and the proportion of patients with and without severe outcome (death) in each cluster/sub-cluster **(B)**. Among the breast cancer samples, the endocrine receptor (estrogen/progesterone) positives are abbreviated as BRER, human epidermal growth factor receptor 2 positives are abbreviated as BRHR, triple positives (estrogen, progesterone, and HER2 receptor positive) are abbreviated as BRTP and the triple negatives (absence of estrogen, progesterone, and HER2 receptors) are abbreviated as BRTN.

For these analyses the hierarchical clustering for each of the four different breast cancer types were further grouped into sub-clusters based on microbial detections (Figure 5A). In the BRTNs the cases of sub-cluster 2b had the highest (63%) proportion of the patients who had died, followed by that of Cluster 1 (33%); while sub-clusters 2a and 2c had a higher number of surviving patients (Figure 5B). The shared feature of sub-clusters 1 and 2b is a higher detection of fungal and parasitic signatures (Figure 5A). BRTP samples did not fall into discrete sub-clusters (Figure 5A), but overall BRTP showed 82% surviving patients. For BRER samples, sub-clusters 1a, b and c had similar numbers of patients who had died (25, 22, and 33%, respectively), while these numbers were much lower for sub-clusters 2a and 2b. Sub-clusters 2a and 2b are notable in that they have an overall more robust and diverse microbial signature. Examining the sub-clusters for BRHR shows a high number of surviving patients in all sub-clusters (1a, 1b, and 2; 75, 86, and 85%, respectively). Within the limits of the data these analyses suggest that the specific microbial signatures may correlate with outcome especially in the case of BRTN.

**Figure 6:**
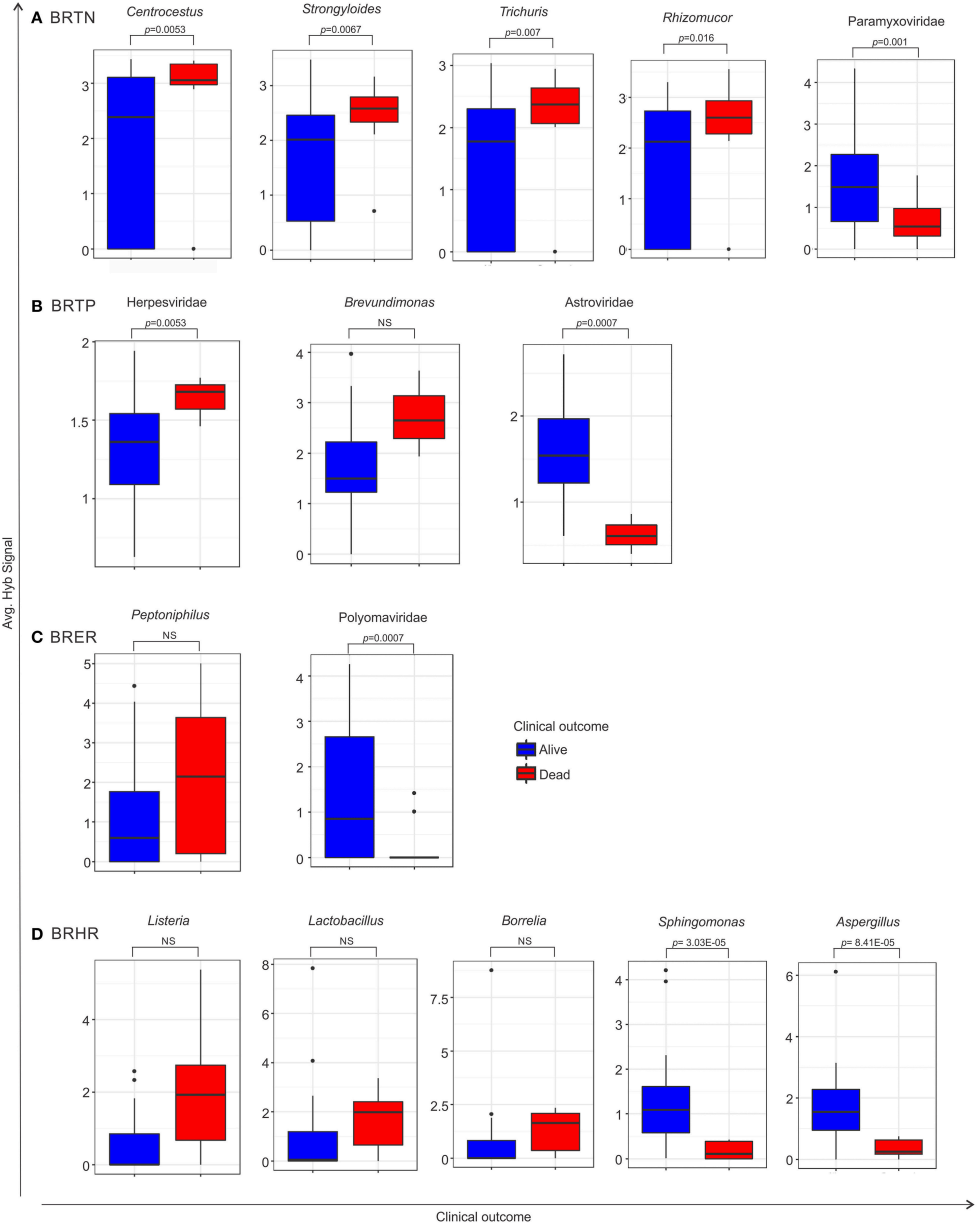
Box plots representing either significant or trend of higher detection of microbial signatures in BRTN **(A)**, BRTP **(B)**, BRER **(C)**, and BRHR **(D)** cases with low (alive) or severe (dead) clinical outcomes, compared by one sided *t*-test. The *p*-value of the tests is shown in the figure if significant. NS represents non-significant test, however still a trend cannot be ignored. Among the breast cancer types, the endocrine receptor (estrogen/progesterone) positives are abbreviated as BRER, human epidermal growth factor receptor 2 positives are abbreviated as BRHR, triple positives (estrogen, progesterone, and HER2 receptor positive) are abbreviated as BRTP and the triple negatives (absence of estrogen, progesterone, and HER2 receptors) are abbreviated as BRTN.

Using the survival data, we also examined variation in average hybridization signal for microbial signatures between the breast cancer types (Figure 6 and Table 6). Interestingly, these analyses showed that high hybridization signals of specific viruses and microbes in a particular breast cancer type may trend with patients who had died, others trended with surviving patients. For example, in BRTP Herpesviridae signatures were detected significantly higher in BRTP patients who had died. Similarly, BRTN patients who had died had significant higher hybridization signals for certain fungal (*Malassezia, Rhizomucor, Rhodotorula*) and parasitic (*Centrocestus, Strongyloides, Trichuris, Contracaecum, Leishmania*) signatures. In the BRERs we found a trend of higher detection of the bacterium *Peptinophilus* signatures in the deceased cases. Similarly, we found a trend of higher detection of certain bacteria (*Listeria, Lactobacillus, Borrelia*) in the BRHR cases with severe outcome.

**Table 6:** One sided *t*-test of microbial signature detection in different breast cancer types [endocrine receptor positives (BRER), human epidermal growth factor receptor 2 positives (BRHR), triple positives (BRTP) and the triple negatives (BRTN)], with their clinical outcome.

**Organism**	***p-*value**	**Adjust *p*-value**	**logFC**
**BRTN_DECEASED_VS_ALIVE_T_TEST**			
*Centrocestus*	0.0053	0.129	0.752
*Strongyloides*	0.00667	0.129	0.546
*Trichuris*	0.00708	0.129	0.745
*Malassezia*	0.0119	0.129	0.701
*Contracaecum*	0.01253	0.129	0.664
*Leishmania*	0.0138	0.129	0.752
*Rhizomucor*	0.01642	0.131	0.631
*Rhodotorula*	0.01881	0.132	0.79
**BRTN_ALIVE_VS_DECEASED_T_TEST**			
Paramyxoviridae	0.00131	0.0733	1.251
Filoviridae	0.00586	0.1642	1.578
*Onchocerca*	0.01925	0.282	1.466
*Orientia*	0.02014	0.282	1.809
*Schistosoma*	0.03627	0.4062	1.393
*Arcobacter*	0.04846	0.4191	1.147
**BRTN_DECEASED_VS_ALIVE_T_TEST**			
Herpesviridae	0.02802	0.9685	0.282
**BRTN_ALIVE_VS_DECEASED_T_TEST**			
Astroviridae	0.00072	0.0512	1.339
Anelloviridae	0.00179	0.0512	3.241
*Campylobacter*	0.00181	0.0512	2.478
*Coccidioides*	0.01974	0.4195	2.257
Hepeviridae	0.0313	0.5018	1.44
*Angiostrongylus*	0.0368	0.5018	2.033
Adenoviridae	0.04206	0.5018	0.327
**BRTN_DECEASED_VS_ALIVE_T_TEST**			
*Peptoniphilus*	0.10238	0.9997	0.879
**BRTN_ALIVE_VS_DECEASED_T_TEST**			
*Eikenella*	0.00027	0.0154	2.179
*Kingella*	0.00047	0.0154	2.111
*Caulobacter*	0.00071	0.0154	2.23
Polyomaviridae	0.00073	0.0154	2.26
*Geotrichum*	0.00089	0.0154	1.362
*Alcaligenes*	0.00146	0.0211	1.735
*Ajellomyces*	0.00206	0.0256	2.114
*Escherichia*	0.00239	0.026	1.465
Circoviridae	0.00405	0.0391	1.203
*Cardiobacterium*	0.00603	0.0485	1.659
*Helicobacter*	0.00715	0.0485	1.869
*Anaplasma*	0.00758	0.0485	1.697
*Arcanobacterium*	0.00762	0.0485	1.135
Herpesviridae	0.00781	0.0485	0.696
**BRTN_DECEASED_VS_ALIVE_T_TEST**			
*Listeria*	0.08097	1	2.008
*Lactobacillus*	0.17059	1	0.811
*Borrelia*	0.20093	1	0.762
**BRTN_ALIVE_VS_DECEASED_T_TEST**			
*Sphingomonas*	3.03*E*−05	0.0021	2.825
*Aspergillus*	8.41*E*−05	0.0029	2.239
*Coxiella*	0.00086	0.02	1.865
*Candida*	0.00188	0.0328	1.154
*Epidermophyton*	0.00306	0.0428	2.234

*Nominal p-value (p-value) and p-value with multiple correction (adjust p-value) for each microbial signature detection along with the log2 fold change (logFC) for the t-tests are mentioned*.

Conversely, high hybridization signals for Paramyxoviridae, Astroviridae, and Polyomaviridae were found with greater frequency, respectively, in the BRTN, BRTP, and BRER cancer patients who survived. Additionally, high hybridization signals for the bacteria *Sphingomonas* and the fungus *Aspergillus* were detected in the BRHR patients who survived. Again within the limits of the clinical data these finding suggest that the qualitative and quantitative nature of the microbial signatures associated with a patient's cancer may provide diagnostic and prognostic information.

### Validation of pathochip screen results by PCR

**Figure 7:**
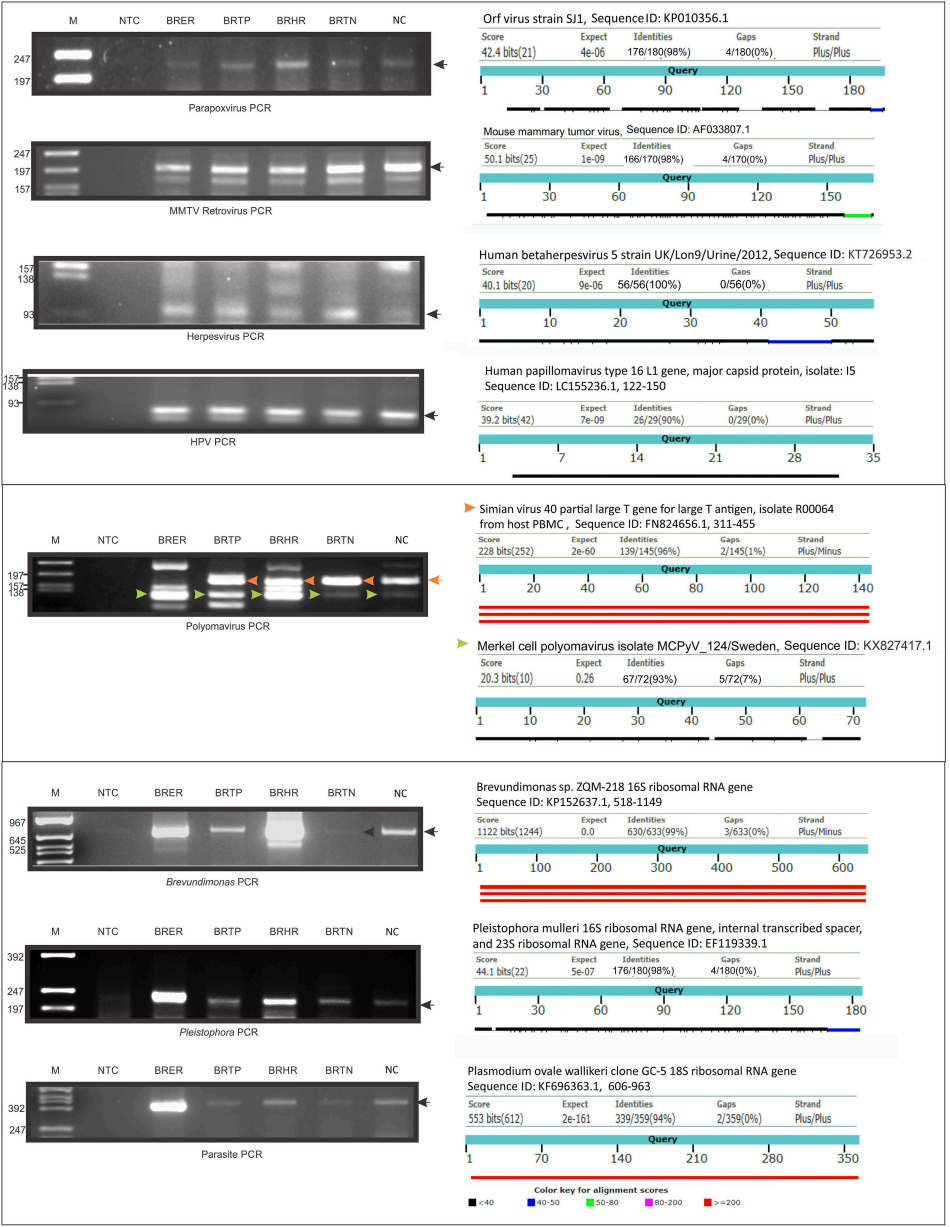
PCR validation of microbial signatures in the 4 types of breast cancers and healthy control, using the primers from Table 7. Among the breast cancer types, the endocrine receptor (estrogen/progesterone) positives are abbreviated as BRER, human epidermal growth factor receptor 2 positives are abbreviated as BRHR, triple positives (estrogen, progesterone, and HER2 receptor positive) are abbreviated as BRTP and the triple negatives (absence of estrogen, progesterone, and HER2 receptors) are abbreviated as BRTN. The breast control samples obtained from healthy individuals are abbreviated as NC. The **left** shows the cropped gel pictures of EtBr stained amplicons run on agarose gel, where M is DNA ladder of RsaI digested ϕX/174, NTC is non-template control. The sequenced amplicons were subjected to nucleotide blast program in NCBI, and the results are shown in the **right**. In the Polyomavirus PCR gel picture, the orange and the green arrow heads signify Simian virus 40 and Merkel cell polyomavirus amplicons respectively, the electropherogram of the sequences of which are marked with the same arrow heads in Supplementary Figure S3.

We selected several viruses and microorganisms detected in the BC samples for verification by non-quantitative PCR and sequencing, these included several viral families and individual viruses (Herpesvirus, Polyoma, Papilloma, Parapox, and MMTV), as well as a prevalent bacterium (*Brevundimonas*), and fungus (*Pleistophora*). The primers used were either previously published (Table 7) or were designed based on sequences from the conserved and specific regions of the micro-organisms. For detection of parasites we used pan-parasite diagnostic PCR primers enabling exhaustive detection of non-human eukaryotic species-specific small subunit rDNA in human clinical samples. For the validation experiments we used the WTA prepared and used for the initial screening. The PCR amplification showed the expected amplicons for the PathoChip-detected viruses, as well as the selected bacterium, fungus and parasite (Figure 7). Sequencing of the PCR products verified the detection of the appropriate virus or other microorganism (Supplementary Table S3, Supplementary Figure S3).

**Table 7:** Primers used for PCR validation of PathoChip screen.

**Micro-organism**	**Primers**	**Sequence (5′-3′)**	**Annealing temp and time**	**Extension temp and time**	**Amplicon size (bp)**	**Detail**
Herpes	FP 1	GAA GAC GCT GAT GAA CCA CG	51°C for 45 s	65°C for 20 s	96	Self-designed
	RP 2	AAG CAC CTG GTG TAC TTT CAC				
Mouse mammary tumor virus (MMTV)	SN FP 5 (gag) RP 6 (gag)	ACT CAG AAG GAA ACC CCT GCC TC ATC TCC TTT TTC CCT GGC CTC TGC	57°C for 30 s	65°C for 30 s	70	Self-designed
HPV	HPV GP5	TTTGTTACTGTGGTAGATACTA	43°C for30 s	65°C for 30 s	104–141	Self-designed
	HPV gp6	GAAAAATAAACTGTAAATCATATTC				
Polyoma	PYV.for PYV.rev	GGAAAGTCTTTAGGGTCTTCTACC TAGGTGCCAACCTATGGAACAGA	53°C for 30 s	65°C for 30 s	178–183	N Engl J Med 1992; 326:988–993 April 9, 1992
Parapox	FP	ATC TTC ACG GGC GCA GTC G	56°C for 30 s	65°C for 30 s	286	Self-designed
	RP	CTC TTC GAC GAC GAC GGG AAC				
Bacteria *Brevundimonas*	FP 17 RP 18	TTG CAG AGG ACA ATC CGA ACT GAG AAC TGC CTT TGA TAC TGG CGA TC	52°C for 30 s	65°C for 60 s	667	Self-designed
Fungus *Pleistophora*	FP 19	AGG TCT CCT AGG TGA ATA GCC	48°C for 30 s	65°C for 30 s	219	Self-designed
	RP 20	CCG TGC TTA CAG TTA TTT CCT C				
Parasite	G3Fl G3Rl	GCCAGCAGCCGCGGTAATTC ACATTCTTGGCAAATGCTTTCGCAG	48°C for 30 s	65°C for 30 s	404	Patents WO 2014071946 A1

## Discussions

The human microbiome is comprised of mutualistic, pathogenic, transient and residential viruses and microorganisms. Many recent studies have suggested that the body's microbiome dramatically affects health, where perturbation of the microbiome leads to altered physiology and pathology, including cancer. However, the reverse may also be true, that different human diseases create disease microenvironments amenable to the persistence of a differential microbiome, with or without a direct effect on the establishment or progression of the disease. Such differential microbiomes could be specific to each such disease. Using our in-house metagenomic array technology (PathoChip), we previously established distinct microbial signatures in triple negative breast cancers (BRTNs) (Banerjee et al., 2015). In the present study we determined the microbial signatures that were significantly higher in the 4 major breast cancer types (BRTN, BRTP, BRER, BRHR) compared to the healthy breast control tissues, and also determined whether the microbial signatures associated with the BRTNs was a specific feature of BRTNs, or a generic feature shared with other types of breast cancers.

Our data showed that the various breast cancers have robust and varied micro-organisms with aspects that are unique to each type as well as shared components. The data suggest that breast cancer microbial signatures may provide type-specific communities of organisms unique to each breast cancer type. We also point out that our control FFPE samples, processed in the same way as tumor samples, had different signatures, generally with much lower hybridization signals, arguing against gross contamination.

Examining viral signatures we found that the majority of the viral families detected were associated with all 4 breast cancer types. However, several important viruses were differentially detected; for example, among known oncogenic viruses the signatures of Polyomaviridae were detected with high significance and high signal intensity in the BRER and BRHR samples and with low signal intensity in the other breast cancer types. Signatures of Hepadnaviridae were similarly detected in BRER and BRTPs with high signal intensity, but with very low signal intensity in the other two cancer types. It is intriguing that signatures for Parapoxviridae family were found in all the breast cancers with BRERs showing the highest level of detection. Parapox viruses are known to have homologs to human genes responsible for angiogenesis (Ueda et al., 2003; Delhon et al., 2004).

There were a number of bacterial families shared by all four breast cancer types. For example, all four breast cancer types had dominant signatures for Proteobacteria followed by Firmicutes. The presence of these two bacterial phyla in the breast cancer tissues has been reported (Urbaniak et al., 2014, 2016; Hieken et al., 2016), and suggested to be a result of adaptation to the fatty acid environment and metabolism in the tissue (Urbaniak et al., 2014). Another study found a positive correlation between Proteobacteria and the metabolic by-products of fatty acid metabolism, along with host-derived genes involved in fatty acid biosynthesis (El Aidy et al., 2013). In particular, the signature of the proteobacteria *Brevundimonas* genus was detected with high hybridization signal and prevalence in all four breast cancer types. *Brevundimonas* causes bacteremia and has been found associated with immunocompromised and/or cancer patients in other studies (Han and Andrade, 2005; Lee et al., 2011; Banerjee et al., 2015). Additionally, the *Mobiluncus* family was detected in all four types, it is mostly known to be associated with bacterial vaginosis (Gatti, 2000); however, the association of breast cancers may correlate with recent studies showing an association with breast abscesses and extragenital infections (Glupczynski et al., 1984; Sturm, 1989). We also detected Actinomyces signatures in all four breast cancers, especially in BRHRs where it was detected with very high signal intensity. Previous studies have reported Actinomycosis in the breast tissues of breast cancer patients (Aamir and Bokhari, 2005; Abdulrahman and Gateley, 2015; Banerjee et al., 2015), as primary (Salmasi et al., 2010), or secondary infections (Brunner et al., 2000) in breast, and in breast abscess (Attar et al., 2007). Additionally, each type of breast cancer held signatures for unique bacterial genera, and may provide an ability to detect specific breast cancer types.

Fungal infections in cancer patients are common. Among the fungal signatures we detected were yeasts like *Candida, Geotrichum, Rhodotorula, Trichosporon* as well as fungi causing Mucormycosis, Aspergillosis (cutaneous infections) and dermatophytes like *Epidermophyton* and *Trichophyton* are commonly known to be associated with cancers (Mays et al., 2006; Ansari et al., 2015; Banerjee et al., 2015; Jung et al., 2015; Rodríguez-Gutiérrez et al., 2015; Berkovits et al., 2016). Also, we detected *Fonsecaea* infection, which is seen to predispose squamous cell carcinoma development (Azevedo et al., 2015).

Possibly the most intriguing and unexpected result of the PathoChip screening is the detection of parasite signatures in different breast cancer types. These signatures were quite unique to the different breast cancer types with no signal parasite being prevalently found in all four. Many parasite signatures were distinctly detected in only one type of breast cancer. It should be kept in mind that our sensitive detection approach allows us to detect low abundance organisms, as well as unknown members of parasite families. However, the association of specific parasites with cancer is known. Among the parasites detected, *Trichinella* (detected in BRTN) has been found in a patient with recurrent ductal invasive breast carcinoma (Kristek et al., 2005). *Schistosoma* (detected in BRTN, BRTP) has been linked to bladder cancer (Samaras et al., 2010; Benamrouz et al., 2012); additionally we detected signatures of *Ascaris* (BRHR, BRER) and *Trichuris* (BRTN) which have been associated with pediatric cancers (Menon et al., 1999). Similarly, *Strongyloides* (BRTN, BRHR) has been associated with adult cancer patients (Guarner et al., 1997). Other signatures detected, *Leishmania* (BNTN) and *Plasmodium* (BRHR, BRTP, BRER), induce the inhibition of apoptosis (Heussler et al., 2001), which may promote oncogenesis (Lowe and Lin, 2000).

It was interesting to further investigate if detection of certain microbial signatures in breast cancers differed among patients who survived or died. We noticed higher detection of certain parasitic and fungal signatures in BRTN patients who died. Of particular interest in these analyses was the finding of high hybridization signals of specific viruses and microbes in a particular breast cancer type that may trend with patients who died, while others trended with surviving patients. Within the limits of the clinical data that could be provided, our findings suggest that the qualitative and quantitative nature of the microbial signatures associated with a patient's cancer may provide diagnostic and prognostic information.

Our findings suggest that the micro-organisms in breast cancers are diverse, extensive and have unique aspects that differentiate the four different breast cancers tested. We represented the microbial signatures that were significantly higher in the breast tumor microenvironment, when compared to healthy breast tissues. Some of these tumor microbial signatures overlapped with the reported skin microbiome (Findley and Grice, 2014; Hieken et al., 2016). For example: Bacteria like, *Lactobacillus, Prevotella, Staphylococcus, Lactococcus, Streptococcus* have been reported earlier as healthy breast skin flora (Hieken et al., 2016; Urbaniak et al., 2016), *Propionibacterium, Corynebacterium* bacteria, and *Malassezia* fungi has been reported to be common skin commensals (Grice and Segre, 2011). Although the detection of those common skin/healthy breast floras in the breast tumor microenvironment in the current study is not surprising, there still exists a breast tumor specific microbiome, which was also reported by other studies (Urbaniak et al., 2014; Xuan et al., 2014).

Many of the microbial signatures that were detected in one or more of the breast cancer types were not detected in the healthy controls, as mentioned in the results section. Most of those micro-organisms were found in earlier studies to be associated with cancer and/or immunocompromised patients (Kontoyianis et al., 1994; Menon et al., 1999; Narikiyo et al., 2004; Aamir and Bokhari, 2005; Kristek et al., 2005; Ramanan et al., 2014; Abdulrahman and Gateley, 2015; Banerjee et al., 2015, 2016).

It is possible that micro-organisms in the breast cancer could contribute to the origin, potentiation or modulation of oncogenesis. However, it is equally possible that the tumor microenvironment provides favorable conditions for specific micro-organisms to persist more readily than in the normal tissue microenvironment. Moreover, due to HIPAA regulations we could not get any information on the type of treatment these breast cancer patients received. Thus, while we can only assume that the samples from some of the patients could be obtained before treatment, others could be receiving treatment already at the time of sample procurement. Especially patients already receiving treatment could be immunocompromised, which further exposes them to a higher infection rate, and thus detecting higher number of micro-organisms from those samples is not surprising.

Our data demonstrate for the first time that the microbial signatures of BRTN and BRTPs are distinct and significantly different from the microbial signatures largely shared by BRER and BRHR. Furthermore, the unique characteristics of the breast cancer associated microbial signatures potentially provide certain tools for specific diagnosis and treatment of these cancers. These findings are hypothesis-generating and needs further investigation to identify a microbial risk signature for the different breast cancer types and potential microbial-based prevention therapies. A complete review of the microbiome in these breast cancers and healthy controls would open up more insight into answering those questions.

## Author contributions

ER and JA conceptualized the study; SB and ER planned the experiments; SB performed the experiments, analyzed part of the data, made tables and figures for the manuscript, wrote the manuscript, with contributions from ER and JA; ZW and TT analyzed the micro-array data; KP provided technical assistance during the experiments; NS and MF were the pathologists who provided and evaluated the samples for identification of breast cancer and controls; AD identified the patients with different breast cancer types for inclusion in the study.

### Conflict of interest statement

The authors declare that the research was conducted in the absence of any commercial or financial relationships that could be construed as a potential conflict of interest.

## References

[B1] Aamir S. Bokhari N. (2005). Actinomycosis of breast. Int. J. Pathol 3:102.

[B2] Abdulrahman G. O. Jr. Gateley C. A. (2015). Primary actinomycosis of the breast caused by *Actinomyces turicensis* with associated Peptoniphilus harei. Breast Dis. 35, 45–47. 10.3233/BD-140381 25095985

[B3] American Cancer Society W. C. O. (2017). Cancer Facts & Figures 2017. Atlanta, GA: American Cancer Society Available online at: http://www.cancer.org/acs/groups/content/@research/documents/document/acspc-047079.pdf

[B4] Ansari Sh. Shirzadi E. Elahi M. (2015). The Prevalence of fungal infections in children with hematologic malignancy in Ali-Asghar Children Hospital between 2005 and 2010. Iran J. Ped. Hematol. Oncol. 5, 1–10. 25914797 PMC4402151

[B5] Attar K. H. Waghorn D. Lyons M. Cunnick G. (2007). Rare species of actinomyces as causative pathogens in breast abscess. Breast J. 13, 501–505. 10.1111/j.1524-4741.2007.00472.x 17760673

[B6] Azevedo C. M. Marques S. G. Santos D. W. Silva R. R. Silva N. F. Santos D. A.. (2015). Squamous cell carcinoma derived from chronic chromoblastomycosis in Brazil. Clin. Infect. Dis. 60, 1500–1504. 10.1093/cid/civ104 25681378

[B7] Baldwin D. A. Feldman M. Alwine J. C. Robertson E. S. (2014). Metagenomic assay for identification of microbial pathogens in tumor tissues. MBio 5:e01714–14. 10.1128/mBio.01714-14 25227467 PMC4172075

[B8] Banerjee S. Peck K. N. Feldman M. D. Schuster M. G. Alwine J. C. Robertson E. S. (2016). Identification of fungal pathogens in a patient with acute myelogenic leukemia using a pathogen detection array technology. Cancer Biol. Ther. 17, 339–345. 10.1080/15384047.2015.1121349 26619325 PMC4910928

[B9] Banerjee S. Tian T. Wei Z. Peck K. N. Shih N. Chalian A. A.. (2017a). Microbial signatures associated with oropharyngeal and oral squamous cell carcinomas. Sci. Rep. 7:4036. 10.1038/s41598-017-03466-6 28642609 PMC5481414

[B10] Banerjee S. Tian T. Wei Z. Shih N. Feldman M. D. Alwine J. C.. (2017b). The ovarian cancer oncobiome. Oncotarget 8, 36225–36245. 10.18632/oncotarget.16717 28410234 PMC5482651

[B11] Banerjee S. Wei Z. Tan F. Peck K. N. Shih N. Feldman M.. (2015). Distinct microbiological signatures associated with triple negative breast cancer. Sci. Rep. 5:15162. 10.1038/srep15162 26469225 PMC4606812

[B12] Benamrouz S. Conseil V. Creusy C. Calderon E. Dei-Cas E. Certad G. (2012). Parasites and malignancies, a review, with emphasis on digestive cancer induced by Cryptosporidium parvum (Alveolata: Apicomplexa). Parasite 19, 101–115. 10.1051/parasite/2012192101 22348213 PMC3671432

[B13] Berkovits C. Toth A. Szenzenstein J. Deak T. Urban E. Gacser A.. (2016). Analysis of oral yeast microflora in patients with oral squamous cell carcinoma. Springerplus 5:1257. 10.1186/s40064-016-2926-6 27536540 PMC4974209

[B14] Brunner S. Graf S. Riegel P. Altwegg M. (2000). Catalase-negative *Actinomyces neuii* subsp. neuii isolated from an infected mammary prosthesis. Int. J. Med. Microbiol. 290, 285–287. 10.1016/S1438-4221(00)80128-9 10959731

[B15] Charrad M. Ghazzali N. Boiteau V. Niknafs A. (2014). NbClust: an r package for determining the relevant number of clusters in a data set. J. Stat. Softw. 61, 1–36. 10.18637/jss.v061.i06

[B16] Chen Y. Wei J. (2015). Identification of pathogen signatures in prostate cancer using RNA-seq. PLoS ONE 10:e0128955. 10.1371/journal.pone.0128955 26053031 PMC4460021

[B17] Delhon G. Tulman E. R. Afonso C. L. Lu Z. De La Concha-Bermejillo A. Lehmkuhl H. D.. (2004). Genomes of the parapoxviruses ORF virus and bovine papular stomatitis virus. J. Virol. 78, 168–177. 10.1128/JVI.78.1.168-177.2004 14671098 PMC303426

[B18] El Aidy S. Derrien M. Merrifield C. A. Levenez F. Dore J. Boekschoten M. V.. (2013). Gut bacteria-host metabolic interplay during conventionalisation of the mouse germfree colon. ISME J. 7, 743–755. 10.1038/ismej.2012.142 23178667 PMC3603396

[B19] Findley K. Grice E. A. (2014). The skin microbiome: a focus on pathogens and their association with skin disease. PLoS Pathog. 10:e1004436. 10.1371/journal.ppat.1004436 25393405 PMC4231143

[B20] Gatti M. (2000). Isolation of Mobiluncus species from the human vagina. Zentralbl. Bakteriol. 289, 869–878. 10.1016/S0934-8840(00)80017-1 10705619

[B21] Glupczynski Y. Labbe M. Crokaert F. Pepersack F. Van Der Auwera P. Yourassowsky E. (1984). Isolation of Mobiluncus in four cases of extragenital infections in adult women. Eur. J. Clin. Microbiol. 3, 433–435. 10.1007/BF02017365 6542001

[B22] Grice E. A. Segre J. A. (2011). The skin microbiome. Nat. Rev. Microbiol. 9, 244–253. 10.1038/nrmicro2537 21407241 PMC3535073

[B23] Guarner J. Matilde-Nava T. Villasenor-Flores R. Sanchez-Mejorada G. (1997). Frequency of intestinal parasites in adult cancer patients in Mexico. Arch. Med. Res. 28, 219–222. 9204612

[B24] Han X. Y. Andrade R. A. (2005). Brevundimonas diminuta infections and its resistance to fluoroquinolones. J Antimicrob Chemother. 55, 853–859. 10.1093/jac/dki139 15883180

[B25] Heussler V. T. Kuenzi P. Rottenberg S. (2001). Inhibition of apoptosis by intracellular protozoan parasites. Int. J. Parasitol. 31, 1166–1176. 10.1016/S0020-7519(01)00271-5 11563357

[B26] Hieken T. J. Chen J. Hoskin T. L. Walther-Antonio M. Johnson S. Ramaker S.. (2016). The microbiome of aseptically collected human breast tissue in benign and malignant disease. Sci. Rep. 6:30751. 10.1038/srep30751 27485780 PMC4971513

[B27] Jung D. S. Farmakiotis D. Jiang Y. Tarrand J. J. Kontoyiannis D. P. (2015). Uncommon candida species fungemia among cancer patients, Houston, Texas, USA. Emerg. Infect. Dis. 21, 1942–1950. 10.3201/eid2111.150404 26488845 PMC4625381

[B28] Kolde R. (2015). pheatmap: Pretty Heatmaps. R package version 1.0.2.

[B29] Kontoyianis D. P. Vartivarian S. Anaissie E. J. Samonis G. Bodey G. P. Rinaldi M. (1994). Infections due to Cunninghamella bertholletiae in patients with cancer: report of three cases and review. Clin. Infect. Dis. 18, 925–928. 10.1093/clinids/18.6.925 8086554

[B30] Kristek J. Marjanovic K. Dmitrovic B. Krajinovic Z. Sakic K. (2005). Trichinella spiralis and breast carcinoma–a case report. Coll. Antropol. 29, 775–777. 16417199

[B31] Lee M. R. Huang Y. T. Liao C. H. Chuang T. Y. Lin C. K. Lee S. W.. (2011). Bacteremia caused by Brevundimonas species at a tertiary care hospital in Taiwan, 2000-2010. Eur. J. Clin. Microbiol. Infect. Dis. 30, 1185–1191. 10.1007/s10096-011-1210-5 21461849

[B32] Lowe S. W. Lin A. W. (2000). Apoptosis in cancer. Carcinogenesis 21, 485–495. 10.1093/carcin/21.3.485 10688869

[B33] Madigan M. P. Ziegler R. G. Benichou J. Byrne C. Hoover R. N. (1995). Proportion of breast cancer cases in the United States explained by well-established risk factors. J. Natl. Cancer Inst. 87, 1681–1685. 10.1093/jnci/87.22.1681 7473816

[B34] Mays S. R. Bogle M. A. Bodey G. P. (2006). Cutaneous fungal infections in the oncology patient: recognition and management. Am. J. Clin. Dermatol. 7, 31–43. 10.2165/00128071-200607010-00004 16489841

[B35] Menon B. S. Abdullah M. S. Mahamud F. Singh B. (1999). Intestinal parasites in Malaysian children with cancer. J. Trop. Pediatr. 45, 241–242. 10.1093/tropej/45.4.241 10467838

[B36] Morales-Sanchez A. Fuentes-Panana E. M. (2014). Human viruses and cancer. Viruses 6, 4047–4079. 10.3390/v6104047 25341666 PMC4213577

[B37] Narikiyo M. Tanabe C. Yamada Y. Igaki H. Tachimori Y. Kato H.. (2004). Frequent and preferential infection of *Treponema denticola, Streptococcus mitis*, and *Streptococcus anginosus* in esophageal cancers. Cancer Sci. 95, 569–574. 10.1111/j.1349-7006.2004.tb02488.x 15245592 PMC11159681

[B38] R Core Team (2015). R: A Language and Environment for Statistical Computing. Vienna: R Foundation for Statistical Computing.

[B39] Ramanan P. Barreto J. N. Osmon D. R. Tosh P. K. (2014). Rothia bacteremia: a 10-year experience at Mayo Clinic, Rochester, Minnesota. J. Clin. Microbiol. 52, 3184–3189. 10.1128/JCM.01270-14 24951810 PMC4313135

[B40] Rodríguez-Gutiérrez G. Carrillo-Casas E. M. Arenas R. Garcia-Mendez J. O. Toussaint S. Moreno-Morales M. E.. (2015). Mucormycosis in a non-hodgkin lymphoma patient caused by Syncephalastrum racemosum: case report and review of literature. Mycopathologia 180, 89–93. 10.1007/s11046-015-9878-1 25736172

[B41] Salmasi A. Asgari M. Khodadadi N. Rezaee A. (2010). Primary actinomycosis of the breast presenting as a breast mass. Breast Care (Basel). 5, 105–107. 10.1159/000301599 20847823 PMC2931045

[B42] Samaras V. Rafailidis P. I. Mourtzoukou E. G. Peppas G. Falagas M. E. (2010). Chronic bacterial and parasitic infections and cancer: a review. J. Infect. Dev. Ctries. 4, 267–281. 10.3855/jidc.819 20539059

[B43] Schnitt S. J. (2010). Classification and prognosis of invasive breast cancer: from morphology to molecular taxonomy. Mod. Pathol. 23(Suppl. 2), S60–S64. 10.1038/modpathol.2010.33 20436504

[B44] Sheflin A. M. Whitney A. K. Weir T. L. (2014). Cancer-promoting effects of microbial dysbiosis. Curr. Oncol. Rep. 16:406. 10.1007/s11912-014-0406-0 25123079 PMC4180221

[B45] Siegel R. L. Miller K. D. Jemal A. (2016). Cancer statistics, 2016. CA Cancer J. Clin. 66, 7–30. 10.3322/caac.21332 26742998

[B46] Sturm A. W. (1989). Mobiluncus species and other anaerobic bacteria in non-puerperal breast abscesses. Eur. J. Clin. Microbiol. Infect. Dis. 8, 789–792. 10.1007/BF02185846 2512148

[B47] Turnbaugh P. J. Ley R. E. Mahowald M. A. Magrini V. Mardis E. R. Gordon J. I. (2006). An obesity-associated gut microbiome with increased capacity for energy harvest. Nature 444, 1027–1031. 10.1038/nature05414 17183312

[B48] Ueda N. Wise L. M. Stacker S. A. Fleming S. B. Mercer A. A. (2003). Pseudocowpox virus encodes a homolog of vascular endothelial growth factor. Virology 305, 298–309. 10.1006/viro.2002.1750 12573575

[B49] Urbaniak C. Cummins J. Brackstone M. Macklaim J. M. Gloor G. B. Baban C. K.. (2014). Microbiota of human breast tissue. Appl. Environ. Microbiol. 80, 3007–3014. 10.1128/AEM.00242-14 24610844 PMC4018903

[B50] Urbaniak C. Gloor G. B. Brackstone M. Scott L. Tangney M. Reid G. (2016). The microbiota of breast tissue and its association with breast cancer. Appl. Environ. Microbiol. 82, 5039–5048. 10.1128/AEM.01235-16 27342554 PMC4968547

[B51] Xuan C. Shamonki J. M. Chung A. Dinome M. L. Chung M. Sieling P. A.. (2014). Microbial dysbiosis is associated with human breast cancer. PLoS ONE 9:e83744. 10.1371/journal.pone.0083744 24421902 PMC3885448

[B52] Yersal O. Barutca S. (2014). Biological subtypes of breast cancer: prognostic and therapeutic implications. World J. Clin. Oncol. 5, 412–424. 10.5306/wjco.v5.i3.412 25114856 PMC4127612

