# Supplementary material for: Distinct Microbial Signatures Associated With Different Breast Cancer Types
Source: Front Microbiol. 2018 May 15;9:951. doi: 10.3389/fmicb.2018.00951 (PMC5962706; doi:10.3389/fmicb.2018.00951)
Supplement: Supplementary file 2 [file Image_1.pdf]

Title: Distinct Microbial signatures Associated with Different Breast Cancer Types

Sagarika Banerjee, Tian Tian, Zhi Wei, Natalie Shih, Michael D Feldman, Kristen N Peck, Angela M DeMichele, James C. Alwine, Erle S. Robertson

Supplementary figure S1.

Average log2 fold change in signal intensity for the significant probes of each of the signatures detected in the different breast cancer types over their respective measurements in control breast samples.

Supplementary figure S1

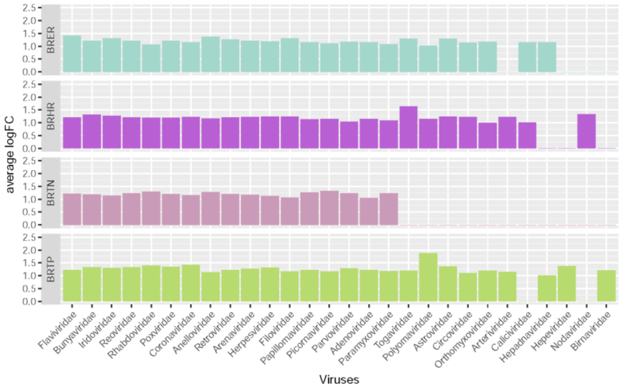

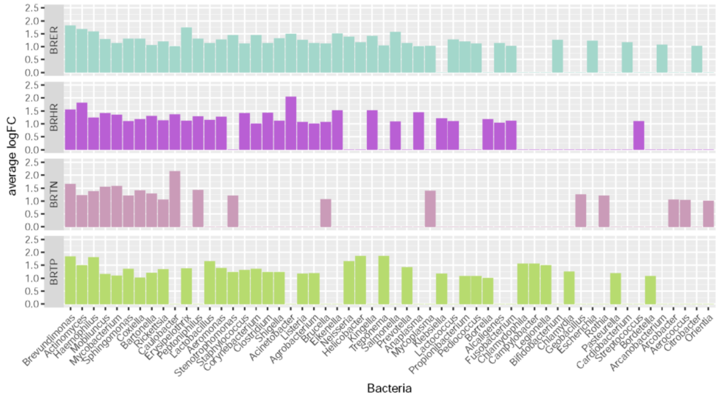

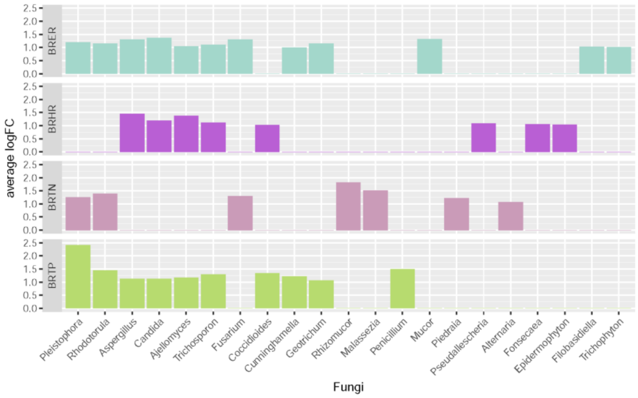

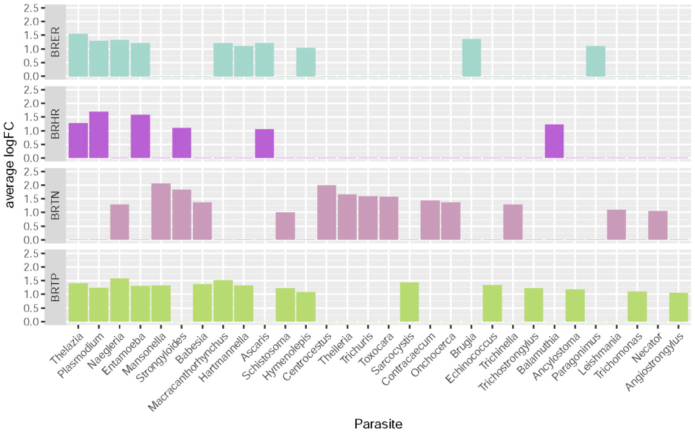

Supplementary figure S2.

Comparative bar and dot plots between two cut-offs; NC (non-matched control) prevalence <= 0.25 and NC prevalence = 0. The bars represent sum of hybridization signal of all the significantly detected probes in each of the cancer types versus the NCs/healthy controls. The dots represent number of probes significantly detected in each of the cancer types versus the NCs/healthy controls. The results of both the cut-offs are not much different. Supplementary figure S2a-d, representing the signatures significantly detected with the above cut-offs in BRER, BRTP, BRHR and BRTN respectively.

Supplementary figure S2a

BRER

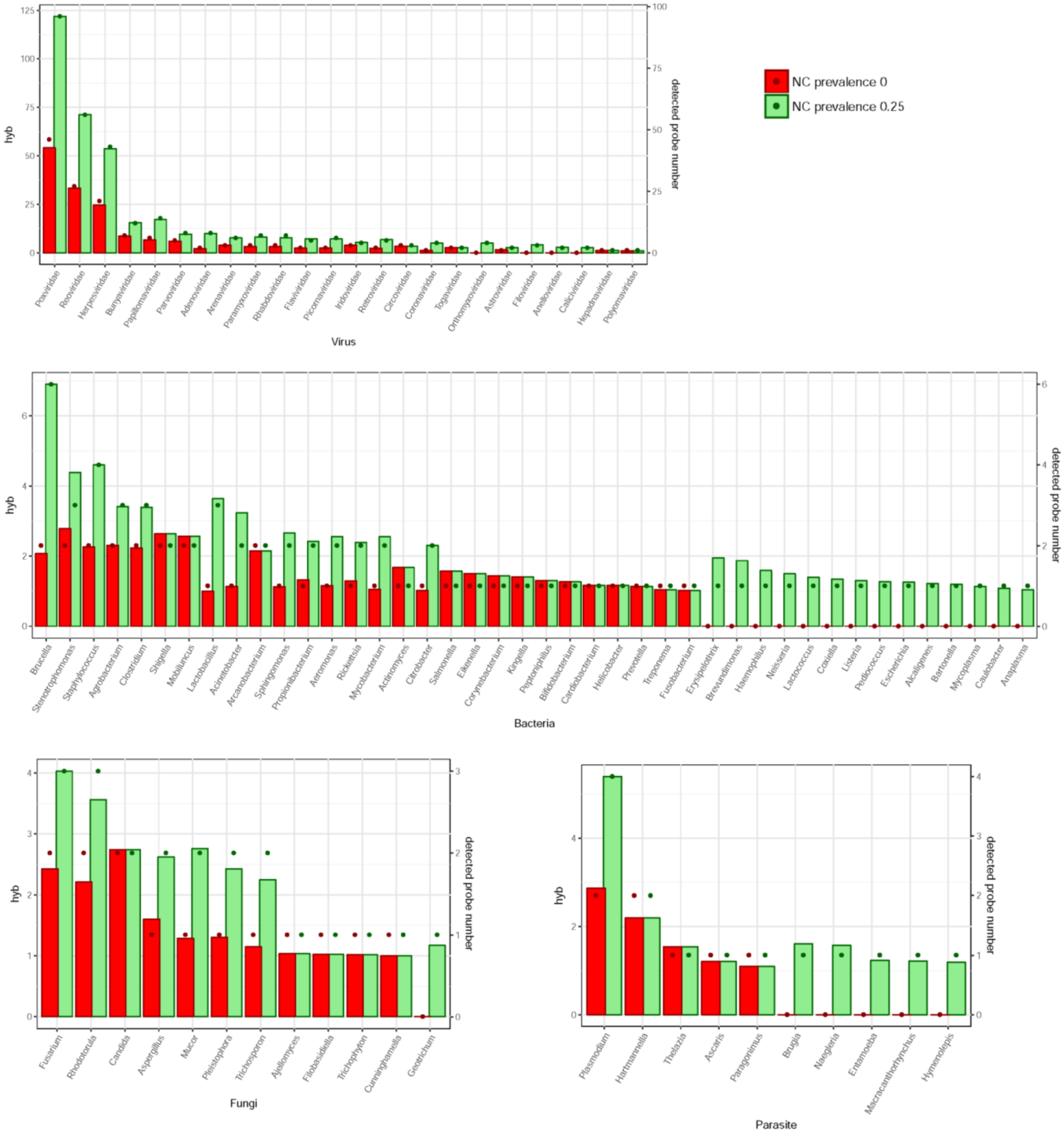

Supplementary figure S2b

BRTP

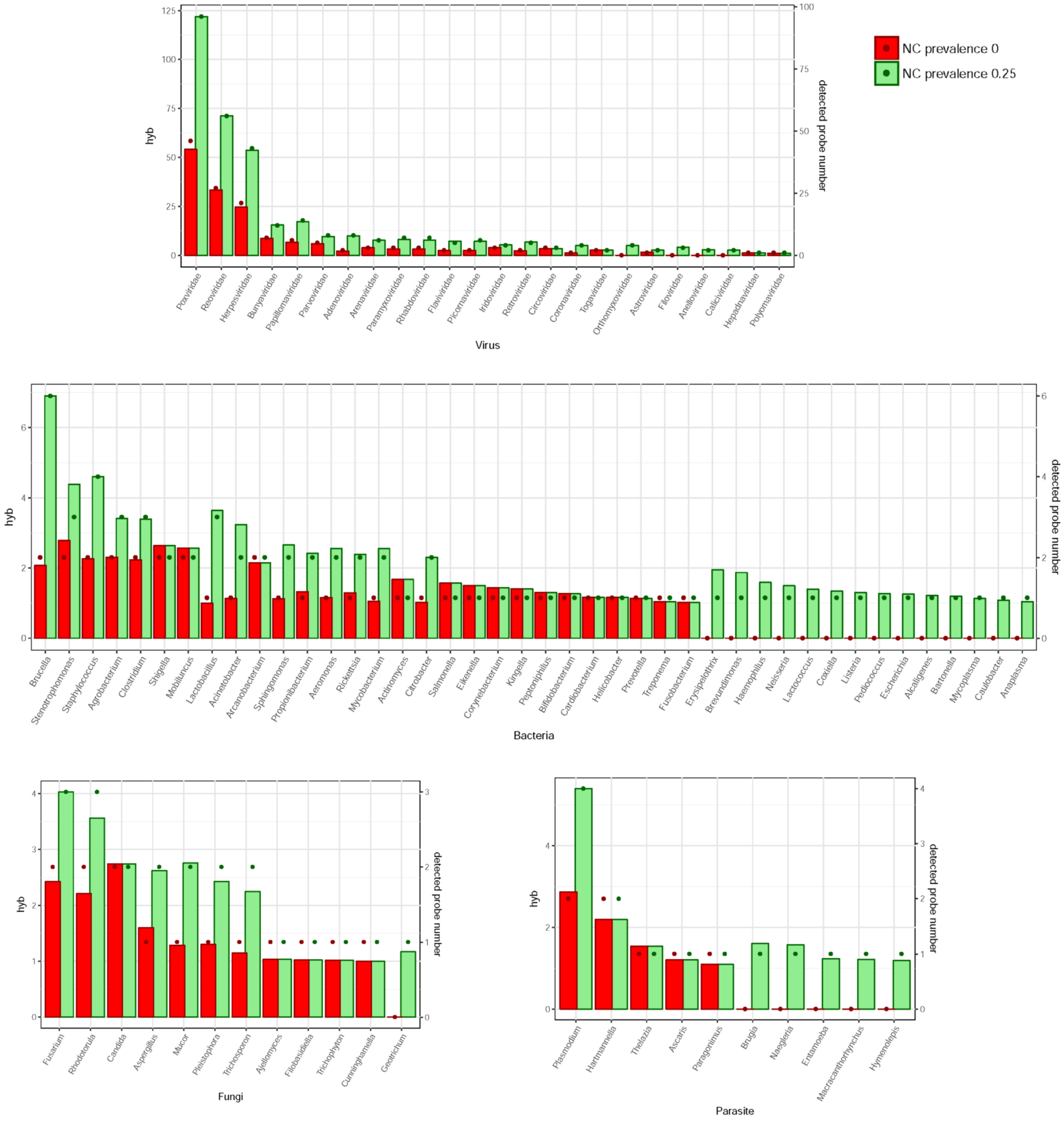

Supplementary figure S2c

BRHR

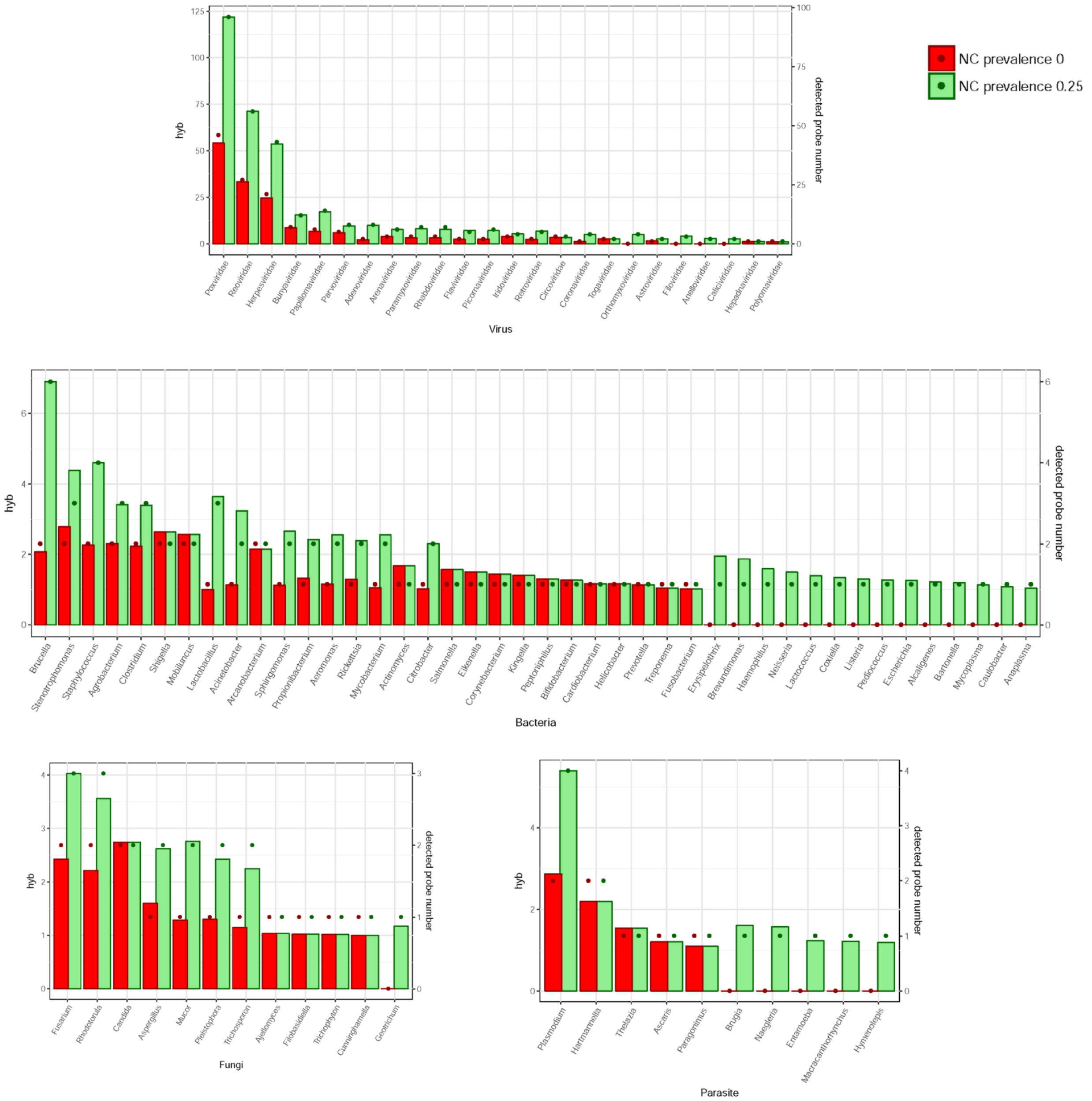

Supplementary figure S2d

BRTN

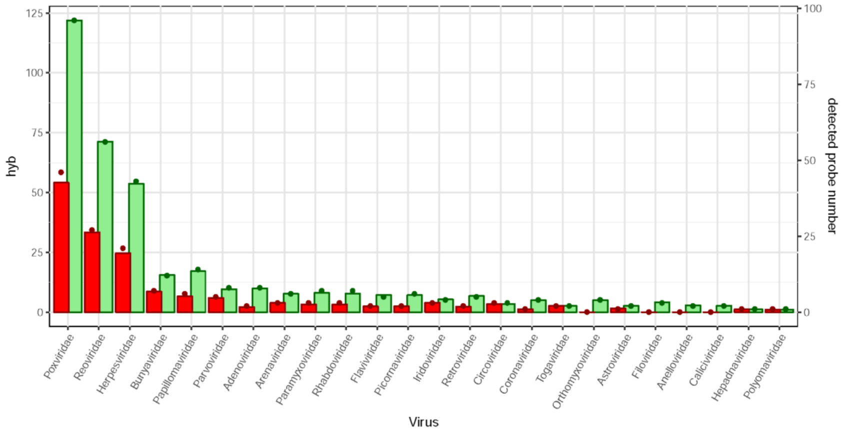

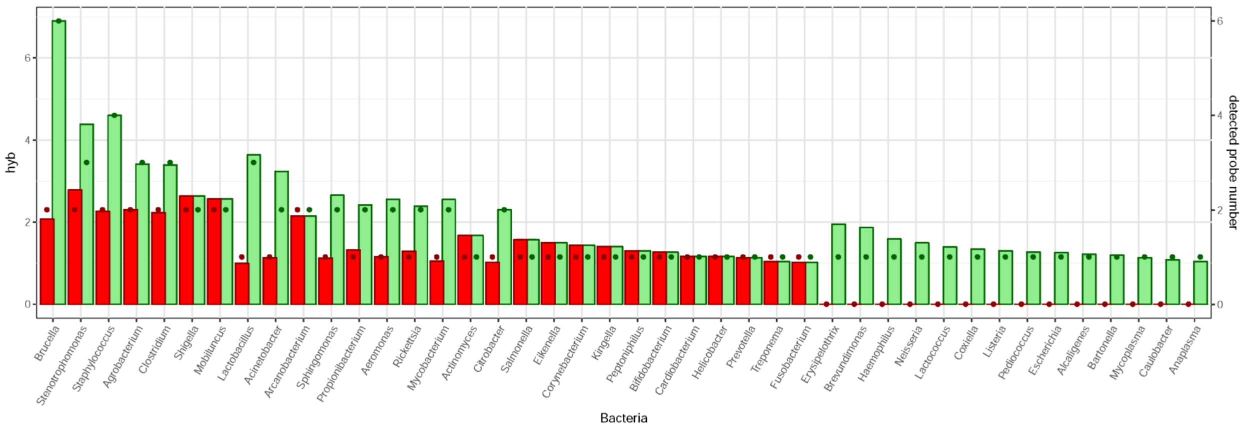

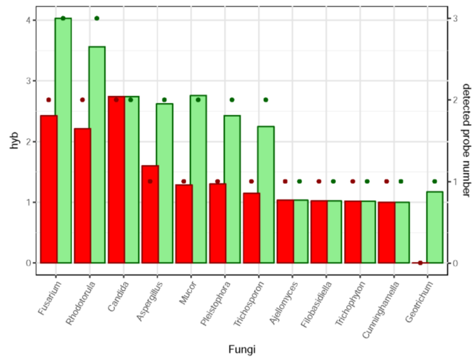

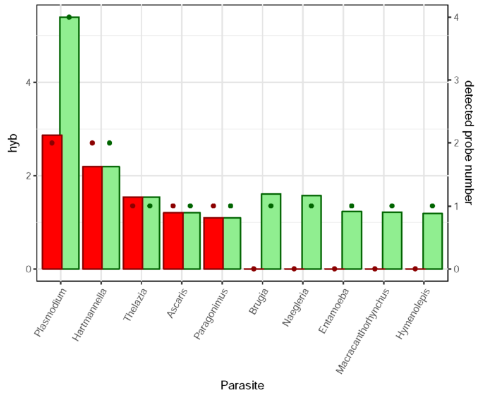

Supplementary Figure S3. Part of the electropherogram of the sequenced amplicons are shown to validate the PathoChip screen results by PCR and Sanger sequencing.

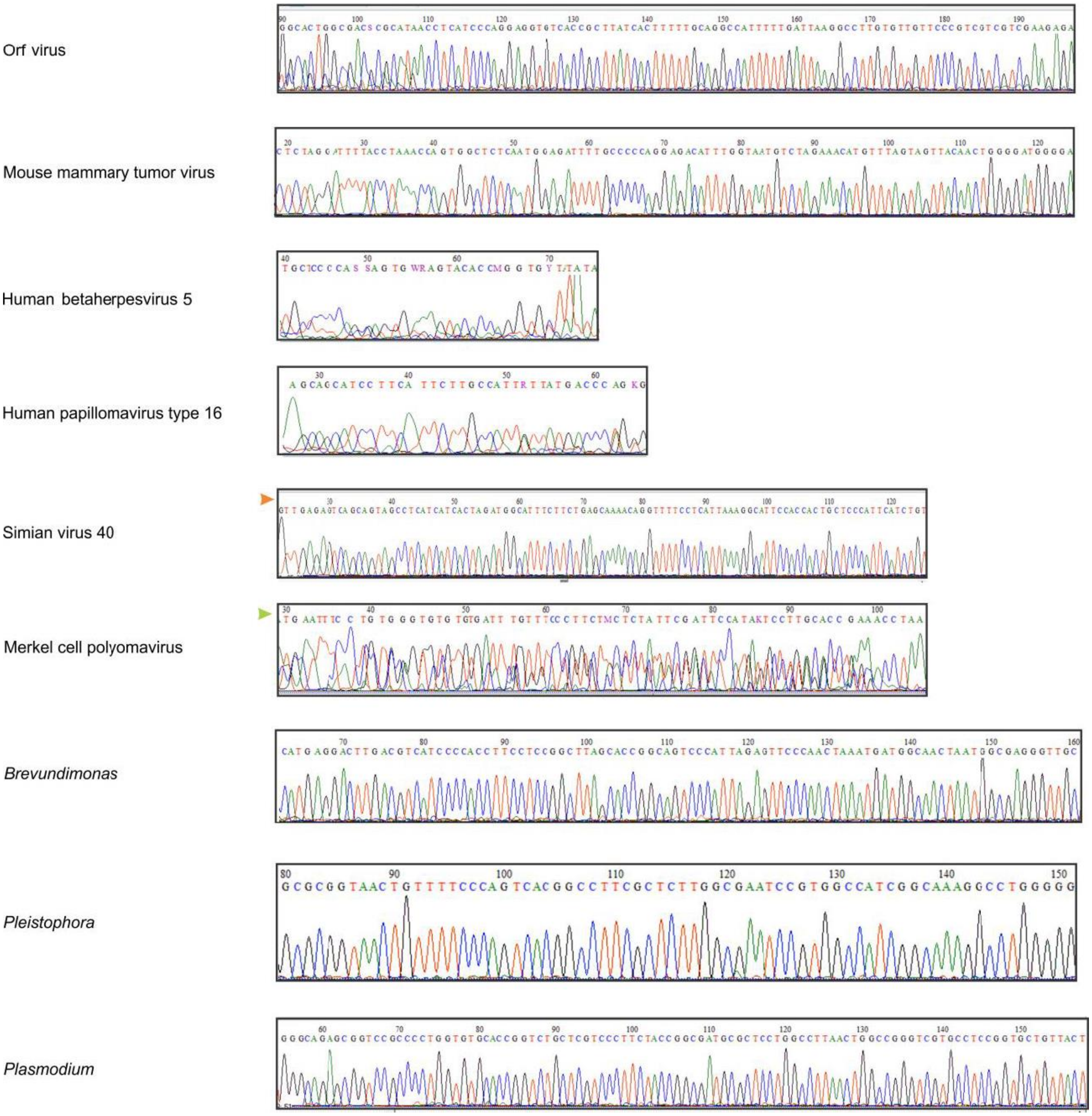

Supplementary table legends.

Supplementary Table S1. Significant detection of the microbial signatures in different breast cancer types by PathoChip screen. The average hybridization signals of the significantly detected probes of micro-organisms in the tumors and in healthy controls are mentioned. The significant detection in the tumors are supported by the adjusted *p*-value <0.01.

Supplementary Table S2. Proportion of probes that were significantly detected (in) and not detected (not in) in each of the the breast cancer types versus the controls.

Supplementary Table S3. BLAST results of the sequenced PCR products for the validation of PathoChip screen.

Supplementary Table S4. Clinical outcomes of the breast cancer patients used in the study.

Supplementary Table S2.

Viral Family	in BRER	not In BRER	in BRHR	not In BRHR	in BRTN	not In BRTN	in BRTP	not In BRTP
Adenoviridae	25%	75%	15%	85%	20%	80%	9%	91%
Anelloviridae	10%	90%	10%	90%	33%	67%	11%	89%
Arenaviridae	8%	92%	8%	92%	10%	90%	13%	88%
Arteriviridae	0%	100%	7%	93%	0%	100%	7%	93%
Astroviridae	13%	87%	8%	92%	0%	100%	10%	90%
Birnaviridae	0%	100%	0%	100%	0%	100%	25%	75%
Bunyaviridae	20%	80%	10%	90%	8%	92%	33%	67%
Circoviridae	100%	0%	25%	75%	0%	100%	25%	75%
Coronaviridae	9%	91%	0%	100%	10%	90%	8%	92%
Filoviridae	14%	86%	7%	93%	0%	100%	10%	90%
Flaviviridae	10%	90%	10%	90%	8%	92%	15%	85%
Hepeviridae	0%	100%	0%	100%	0%	100%	14%	86%
Herpesviridae	15%	85%	15%	85%	8%	92%	17%	83%
Iridoviridae	8%	92%	8%	92%	6%	94%	8%	92%
Nodaviridae	0%	100%	33%	67%	0%	100%	0%	100%
Orthomyxoviridae	25%	75%	25%	75%	0%	100%	25%	75%
Papillomaviridae	14%	86%	13%	87%	14%	86%	18%	82%
Paramyxoviridae	8%	92%	9%	91%	8%	92%	23%	77%
Parvoviridae	50%	50%	33%	67%	8%	92%	100%	0%
Picornaviridae	11%	89%	11%	89%	11%	89%	11%	89%
Polyomaviridae	7%	93%	8%	92%	0%	100%	8%	92%
Poxviridae	13%	87%	13%	87%	17%	83%	9%	91%
Reoviridae	33%	67%	100%	0%	20%	80%	100%	0%
Retroviridae	17%	83%	13%	88%	8%	92%	15%	85%
Rhabdoviridae	13%	88%	13%	88%	8%	92%	13%	88%
Togaviridae	8%	92%	7%	93%	0%	100%	20%	80%
**Bacterial Genera**	**in BRER**	**not In BRER**	**in BRHR**	**not In BRHR**	**in BRTN**	**not In BRTN**	**in BRTP**	**not In BRTP**
Acinetobacter	50%	50%	50%	50%	0%	100%	0%	100%
Actinomyces	50%	50%	50%	50%	50%	50%	50%	50%
Aeromonas	100%	0%	100%	0%	0%	100%	8%	92%
Agrobacterium	15%	85%	8%	92%	0%	100%	8%	92%
Alcaligenes	33%	67%	33%	67%	0%	100%	0%	100%
Anaplasma	8%	92%	10%	90%	0%	100%	0%	100%
Arcanobacterium	100%	0%	0%	100%	0%	100%	0%	100%
Arcobacter	0%	100%	0%	100%	8%	92%	0%	100%
Bartonella	50%	50%	50%	50%	50%	50%	50%	50%
Bifidobacterium	8%	92%	0%	100%	0%	100%	0%	100%
Bordetella	0%	100%	0%	100%	0%	100%	8%	92%
Borrelia	0%	100%	8%	92%	0%	100%	8%	92%
Brevundimonas	100%	0%	100%	0%	33%	67%	100%	0%
Brucella	100%	0%	100%	0%	50%	50%	0%	100%

Campylobacter	0%	100%	0%	100%	0%	100%	8%	92%
Cardiobacterium	33%	67%	0%	100%	0%	100%	0%	100%
Caulobacter	33%	67%	33%	67%	33%	67%	0%	100%
Chlamydia	0%	100%	0%	100%	0%	100%	15%	85%
Chlamydophila	0%	100%	0%	100%	0%	100%	8%	92%
Citrobacter	100%	0%	0%	100%	0%	100%	0%	100%
Clostridium	8%	92%	8%	92%	0%	100%	15%	85%
Corynebacterium	8%	92%	8%	92%	0%	100%	8%	92%
Coxiella	8%	92%	15%	85%	8%	92%	8%	92%
Eikenella	100%	0%	100%	0%	0%	100%	0%	100%
Erysipelothrix	100%	0%	100%	0%	0%	100%	100%	0%
Escherichia	9%	91%	0%	100%	0%	100%	0%	100%
Fusobacterium	8%	92%	8%	92%	0%	100%	0%	100%
Geobacillus	0%	100%	0%	100%	50%	50%	0%	100%
Haemophilus	8%	92%	8%	92%	8%	92%	8%	92%
Helicobacter	8%	92%	0%	100%	0%	100%	8%	92%
Kingella	100%	0%	100%	0%	0%	100%	0%	100%
Klebsiella	0%	100%	8%	92%	0%	100%	8%	92%
Lactobacillus	20%	80%	8%	92%	0%	100%	8%	92%
Lactococcus	8%	92%	8%	92%	0%	100%	0%	100%
Legionella	0%	100%	0%	100%	0%	100%	8%	92%
Listeria	8%	92%	8%	92%	0%	100%	8%	92%
Mobiluncus	100%	0%	100%	0%	100%	0%	100%	0%
Mycobacterium	15%	85%	15%	85%	8%	92%	0%	100%
Mycoplasma	8%	92%	0%	100%	8%	92%	0%	100%
Neisseria	8%	92%	0%	100%	0%	100%	8%	92%
Orientia	0%	100%	0%	100%	8%	92%	0%	100%
Pasteurella	0%	100%	0%	100%	0%	100%	8%	92%
Pediococcus	8%	92%	0%	100%	0%	100%	15%	85%
Peptoniphilus	100%	0%	100%	0%	50%	50%	0%	100%
Prevotella	20%	80%	0%	100%	0%	100%	20%	80%
Propionibacterium	100%	0%	0%	100%	0%	100%	100%	0%
Rickettsia	8%	92%	23%	77%	8%	92%	15%	85%
Rothia	0%	100%	0%	100%	33%	67%	0%	100%
Salmonella	8%	92%	8%	92%	0%	100%	0%	100%
Shigella	8%	92%	8%	92%	0%	100%	15%	85%
Sphingomonas	15%	85%	8%	92%	8%	92%	15%	85%
Staphylococcus	31%	69%	8%	92%	0%	100%	15%	85%
Stenotrophomonas	23%	77%	0%	100%	23%	77%	15%	85%
Streptococcus	0%	100%	10%	90%	0%	100%	0%	100%
Treponema	100%	0%	0%	100%	0%	100%	100%	0%
**Fungal Genera**	**in BRER**	**not In BRER**	**in BRHR**	**not In BRHR**	**in BRTN**	**not In BRTN**	**in BRTP**	**not In BRTP**
Ajellomyces	8%	92%	8%	92%	0%	100%	15%	85%

Aspergillus	33%	67%	33%	67%	0%	100%	0%	100%
Candida	15%	85%	15%	85%	0%	100%	15%	85%
Coccidioides	0%	100%	8%	92%	0%	100%	8%	92%
Cunninghamella	33%	67%	0%	100%	0%	100%	33%	67%
Epidermophyton	0%	100%	8%	92%	0%	100%	0%	100%
Filobasidiella	50%	50%	0%	100%	0%	100%	0%	100%
Fonsecaea	0%	100%	25%	75%	0%	100%	0%	100%
Fusarium	17%	83%	0%	100%	8%	92%	0%	100%
Geotrichum	100%	0%	0%	100%	0%	100%	100%	0%
Malassezia	0%	100%	0%	100%	100%	0%	0%	100%
Mucor	100%	0%	0%	100%	0%	100%	0%	100%
Penicillium	0%	100%	0%	100%	0%	100%	8%	92%
Piedraia	0%	100%	0%	100%	33%	67%	0%	100%
Pleistophora	100%	0%	0%	100%	100%	0%	100%	0%
Pseudallescheria	0%	100%	20%	80%	0%	100%	0%	100%
Rhizomucor	0%	100%	0%	100%	25%	75%	0%	100%
Rhodotorula	100%	0%	0%	100%	33%	67%	100%	0%
Trichophyton	100%	0%	0%	100%	0%	100%	0%	100%
Trichosporon	50%	50%	50%	50%	0%	100%	20%	80%
**Parasitic Genera**	**in BRER**	**not In BRER**	**in BRHR**	**not In BRHR**	**in BRTN**	**not In BRTN**	**in BRTP**	**not In BRTP**
Ancylostoma	0%	100%	0%	100%	0%	100%	13%	88%
Angiostrongylus	0%	100%	0%	100%	0%	100%	100%	0%
Ascaris	100%	0%	100%	0%	0%	100%	0%	100%
Babesia	0%	100%	0%	100%	50%	50%	50%	50%
Balamuthia	0%	100%	50%	50%	0%	100%	0%	100%
Brugia	100%	0%	0%	100%	0%	100%	0%	100%
Centrocestus	0%	100%	0%	100%	50%	50%	0%	100%
Contracaecum	0%	100%	0%	100%	100%	0%	0%	100%
Echinococcus	0%	100%	0%	100%	0%	100%	14%	86%
Entamoeba	20%	80%	20%	80%	0%	100%	20%	80%
Hartmannella	50%	50%	0%	100%	0%	100%	50%	50%
Hymenolepis	100%	0%	0%	100%	0%	100%	33%	67%
Leishmania	0%	100%	0%	100%	33%	67%	0%	100%
Macracanthorhynchus	50%	50%	0%	100%	0%	100%	50%	50%
Mansonella	0%	100%	0%	100%	50%	50%	100%	0%
Naegleria	100%	0%	0%	100%	8%	92%	20%	80%
Necator	0%	100%	0%	100%	33%	67%	0%	100%
Onchocerca	0%	100%	0%	100%	100%	0%	0%	100%
Paragonimus	50%	50%	0%	100%	0%	100%	0%	100%
Plasmodium	100%	0%	100%	0%	0%	100%	100%	0%
Sarcocystis	0%	100%	0%	100%	0%	100%	100%	0%
Schistosoma	0%	100%	0%	100%	8%	92%	7%	93%
Strongyloides	0%	100%	17%	83%	33%	67%	0%	100%

Theileria	0%	100%	0%	100%	20%	80%	0%	100%
Thelazia	100%	0%	100%	0%	0%	100%	100%	0%
Toxocara	0%	100%	0%	100%	100%	0%	0%	100%
Trichinella	0%	100%	0%	100%	14%	86%	0%	100%
Trichomonas	0%	100%	0%	100%	0%	100%	100%	0%
Trichostrongylus	0%	100%	0%	100%	0%	100%	100%	0%
Trichuris	0%	100%	0%	100%	14%	86%	0%	100%

Supplementary Table S3. BLAST results of the sequenced PCR products for the validation of PathoChip screen.

Micro-organism	Primers used	Sequence BLAST Results
MMTV	SN FP 5 and RP 6 (gag)	Mouse mammary tumor virus gag gene (AF033807.1),query coverage 98%<br><br>Human mammary tumor virus gag-pro-pol precursor protein gene (AF248269.1), query coverage 97%
HPV	HPV GP5 and HPV gp6	Human papillomavirus type 16 L1 gene, major capsid protein, isolate: I5 (LC155236.1), query coverage 90%<br><br>Human papillomavirus type 6 L1 gene (LC155239.1), query coverage 100%<br><br>Human papillomavirus isolate H218 (KR674075.1), query coverage 100%
Polyoma	PYV.for and PYV.rev	Merkel cell polyomavirus isolate MCPyV_124/Sweden (KX827417.1) for the 130bp band marked in yellow in figure 5, query coverage 93%<br><br>Simian virus 40 partial large T antigen gene, isolate R00064 (FN824656.1) for the 178bp band marked in orange in figure 5, query coverage 100%
Parapox	FP and RP	Orf virus strain SJ1 (KP010356.1), ORFV072 gene, query coverage 98%
Herpes	FP1 and RP2	Human betaherpesvirus 5 strain UK/Lon9/Urine/2012 (KT726953.2), query coverage 100%
Bacteria *Brevundimonas*	FP 17 and RP 18	Brevundimonas sp. ZQM-218 16S ribosomal RNA gene (KP152637.1), query coverage 100%
Fungus *Pleistophora*	FP 19 and RP 20	Pleistophora mulleri 16S ribosomal RNA gene (EF119339.1), query coverage 98%
Parasite	G3Fl and G3Rl	*Plasmodium ovale* wallikeri clone GC-5 (KF696363.1), query coverage 100%

Supplementary Table S4.

Cancer types	Patient Outcome	Cancer types	Patient Outcome	Cancer types	Patient Outcome	Cancer types	Patient Outcome
BRER 01	Alive	BRTP 01	Deceased	BRHR 01	Alive	BRTN 01	Alive
BRER 02	Alive	BRTP 02	Alive	BRHR 02	Alive	BRTN 02	Alive
BRER 03	Alive	BRTP 03	Alive	BRHR 03	Alive	BRTN 03	Deceased
BRER 04	Alive	BRTP 04	Alive	BRHR 04	Alive	BRTN 04	Alive
BRER 05	Alive	BRTP 05	Alive	BRHR 05	Alive	BRTN 05	Alive
BRER 06	Alive	BRTP 06	Alive	BRHR 06	Alive	BRTN 06	Deceased
BRER 07	Alive	BRTP 07	Deceased	BRHR 07	Alive	BRTN 07	Alive
BRER 08	Alive	BRTP 08	Alive	BRHR 08	Alive	BRTN 08	Alive
BRER 09	Deceased	BRTP 09	Alive	BRHR 09	Alive	BRTN 09	Alive
BRER 10	Alive	BRTP 10	Alive	BRHR 10	Alive	BRTN 10	Alive
BRER 11	Alive	BRTP 11	Alive	BRHR 11	Deceased	BRTN 11	Alive
BRER 12	Alive	BRTP 12	Deceased	BRHR 12	Alive	BRTN 12	Alive
BRER 13	Alive	BRTP 13	Alive	BRHR 13	Deceased	BRTN 13	Deceased
BRER 14	Alive	BRTP 14	Alive	BRHR 14	Alive	BRTN 14	Deceased
BRER 15	Alive	BRTP 15	Alive	BRHR 15	Alive	BRTN 15	Deceased
BRER 16	Deceased	BRTP 16	Alive	BRHR 16	Alive	BRTN 16	Deceased
BRER 17	Alive	BRTP 17	Alive	BRHR 17	Deceased	BRTN 17	Alive
BRER 18	Alive	BRTP 18	Alive	BRHR 18	Alive	BRTN 18	Deceased
BRER 19	Alive	BRTP 19	Alive	BRHR 19	Deceased	BRTN 19	Alive
BRER 20	Alive	BRTP 20	Alive	BRHR 20	Deceased	BRTN 20	Alive
BRER 21	Alive	BRTP 21	Alive	BRHR 21	Alive	BRTN 21	Alive
BRER 22	Alive	BRTP 22	Alive	BRHR 22	Alive	BRTN 22	Alive
BRER 23	Alive			BRHR 23	Alive	BRTN 23	Deceased
BRER 24	Deceased			BRHR 24	Alive	BRTN 24	Alive
BRER 25	Deceased			BRHR 25	Alive	BRTN 25	Alive
BRER 26	Alive			BRHR 26	Alive	BRTN 26	Alive
BRER 27	Alive			BRHR 27	Alive	BRTN 27	Alive
BRER 28	Alive			BRHR 28	Alive	BRTN 28	Alive
BRER 29	Deceased			BRHR 29	Alive	BRTN 29	Alive
BRER 30	Deceased					BRTN 30	Alive
BRER 31	Alive					BRTN 31	Alive
BRER 32	Alive					BRTN 32	Deceased
BRER 33	Alive					BRTN 33	Deceased
BRER 34	Alive					BRTN 34	Deceased
BRER 35	Alive					BRTN 35	Alive
BRER 36	Alive						
BRER 37	Alive						
BRER 38	Alive						
BRER 39	Deceased						
BRER 40	Deceased						
BRER 41	Alive						
BRER 42	Alive						
BRER 43	Deceased						
BRER 44	Alive						
BRER 45	Alive						
